# Sustained‐Release Photothermal Microneedles for Postoperative Incisional Analgesia and Wound Healing via Hydrogen Therapy

**DOI:** 10.1002/advs.202503698

**Published:** 2025-06-23

**Authors:** Aining Zhang, Xue Jiang, Bingrui Xiong, Jiayi Chen, Xin Liu, Siyuan Wang, Bofu Li, Mian Peng, Wei Li

**Affiliations:** ^1^ Department of Anesthesiology Zhongnan Hospital of Wuhan University Wuhan 430071 P. R. China; ^2^ Department of Burns Tongren Hospital of Wuhan University (Wuhan Third Hospital) School of Pharmaceutical Sciences Wuhan University Wuhan 430071 P. R. China; ^3^ TaiKang Center for Life and Medical Sciences Wuhan University Wuhan 430071 P. R. China; ^4^ Hubei Provincial Key Laboratory of Developmentally Originated Disease Wuhan 430071 P. R. China

**Keywords:** hydrogen, microneedle, personalized analgesia, photothermal excitation, postoperative pain

## Abstract

Effective management of postoperative pain and wound healing presents significant challenges in clinical settings, driving the need for innovative therapeutic approaches. The analgesic and wound healing effects of hydrogen (H_2_) have gradually been recognized; however, the lack of efficient hydrogen delivery systems remains a major limitation. This study introduces a novel transdermal drug delivery system, which utilizes sustained‐release photothermal microneedles (MNs) to ameliorate incisional pain and accelerate wound healing. Polydopamine (PDA)‐modified ZIF‐8@ammonia borane (AB) nanoparticles with photothermal conversion properties are designed, along with temperature‐responsive QX‐314‐loaded polycaprolactone (PCL) microspheres for controlled release, which are delivered in vivo by dissolvable MNs. In vitro results showed that PDA@ZIF‐8@AB nanoparticles can release H_2_ continuously for up to 5 days in an acidic microenvironment, while the photothermal properties of PDA facilitated controlled release of QX‐314 through 6 cycles of near‐infrared (NIR) exposure. In vivo experiments demonstrated that the MN system provided sustained analgesia for up to 5 days and promoted wound healing in the acidic microenvironment of postoperative incisions. Upon NIR exposure, the photothermal conversion of PDA activated membrane ion channels and induced thermally triggered deformation of PCL@QX‐314 microspheres, allowing for on‐demand release of QX‐314 and targeted neuronal uptake, thus offering personalized analgesia. In vitro cell experiments and in vivo studies confirmed the biocompatibility of the system. This innovative approach not only highlights the dual role of H_2_ in pain relief and wound healing but also provides a new personalized treatment strategy for postoperative pain management with promising clinical applications.

## Graphical abstract

This study introduces a novel transdermal delivery system of molecular hydrogen for postoperative pain relief and wound healing. This system utilizes sustained‐release photothermal microneedles to integrate hydrogen‐releasing nanoparticles and temperature‐responsive microspheres, achieving acid‐responsive hydrogen release and controlled drug release. The innovative approach offers sustained analgesia, individualized pain treatment, and promotion of wound healing through multi‐faceted therapeutic mechanisms.

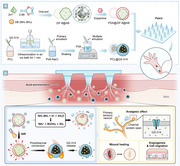

## 1 Introduction

Postoperative pain management is a critical issue that needs to be addressed in modern anesthesiology and perioperative medicine.^[^ ^1^ ^]^ Studies have shown that over 80% of surgical patients experience acute pain during the postoperative period, and ≈10% of patients develop chronic pain due to inadequate management of acute pain.^[^ ^2^ ^]^ Poor pain control not only complicates recovery, potentially prolongs hospital stays, but also leads to delayed wound healing, resulting in impairments of quality of life.^[^ ^3^ ^]^ In clinical practice, local anesthetics are most commonly used to manage postoperative pain, because of their effective analgesic performance and minimal side effects.^[^ ^4^ ^]^ However, the short duration of action following a single administration has hindered the application of local anesthetics in postoperative pain managements.^[^ ^5^ ^]^

In recent years, researchers have begun to explore effective sustained‐release analgesic strategies and innovative drug delivery systems. Among these innovations, liposomal formulations^[^ ^6^ ^]^ and hydrogels^[^ ^7^ ^]^ have been developed to extend the duration of pain relief. Our previous study has also investigated a pH‐responsive self‐monitoring microneedle (MN), which can provide controlled drug release tailored to individual patient needs and effective postoperative analgesia for up to 72 h.^[^ ^8^ ^]^ However, existing MN systems still face challenges related to drug loading capacity and precision of drug release, which impede the clinical translation. Therefore, there is an urgent need to develop new analgesia system to postoperative pain managements.

Recently, hydrogen (H_2_), a colorless, odorless, non‐toxic, renewable gas, has garnered attention for its potential medical applications.^[^ ^9^ ^]^ For instance, H_2_ has demonstrated potential in alleviating various types of pain, including postoperative and neuropathic pain,^[^ ^10^ ^]^ by targeting oxidative stress and inflammatory pathways, offering a promising non‐traditional analgesic option.^[^ ^11^ ^]^ A study by Li et al.^[^ ^12^ ^]^ confirmed that H_2_ could be used as a therapeutic agent to alleviate postoperative pain through the Trx1/ASK1/MMP9 signaling pathway. Additionally, H_2_ has been also reported to promote wound healing and tissue regeneration by reducing inflammation and oxidative stress.^[^ ^13^ ^]^ Therefore, we hypothesize that H_2_ could serve a dual role in postoperative incision management, providing both analgesic effects and promoting healing. However, delivering H_2_ in a controlled and sustained manner still presents challenges, particularly in the acidic microenvironment of postoperative wounds. Current methods, such as H_2_ inhalation, oral intake of H_2_‐rich water, or injection of H_2_ solutions, often require frequent dosing, which can reduce patient compliance and lack the precision needed to maintain localized therapeutic concentrations at surgical sites over extended periods.^[^ ^14^ ^]^

**Figure 1:**
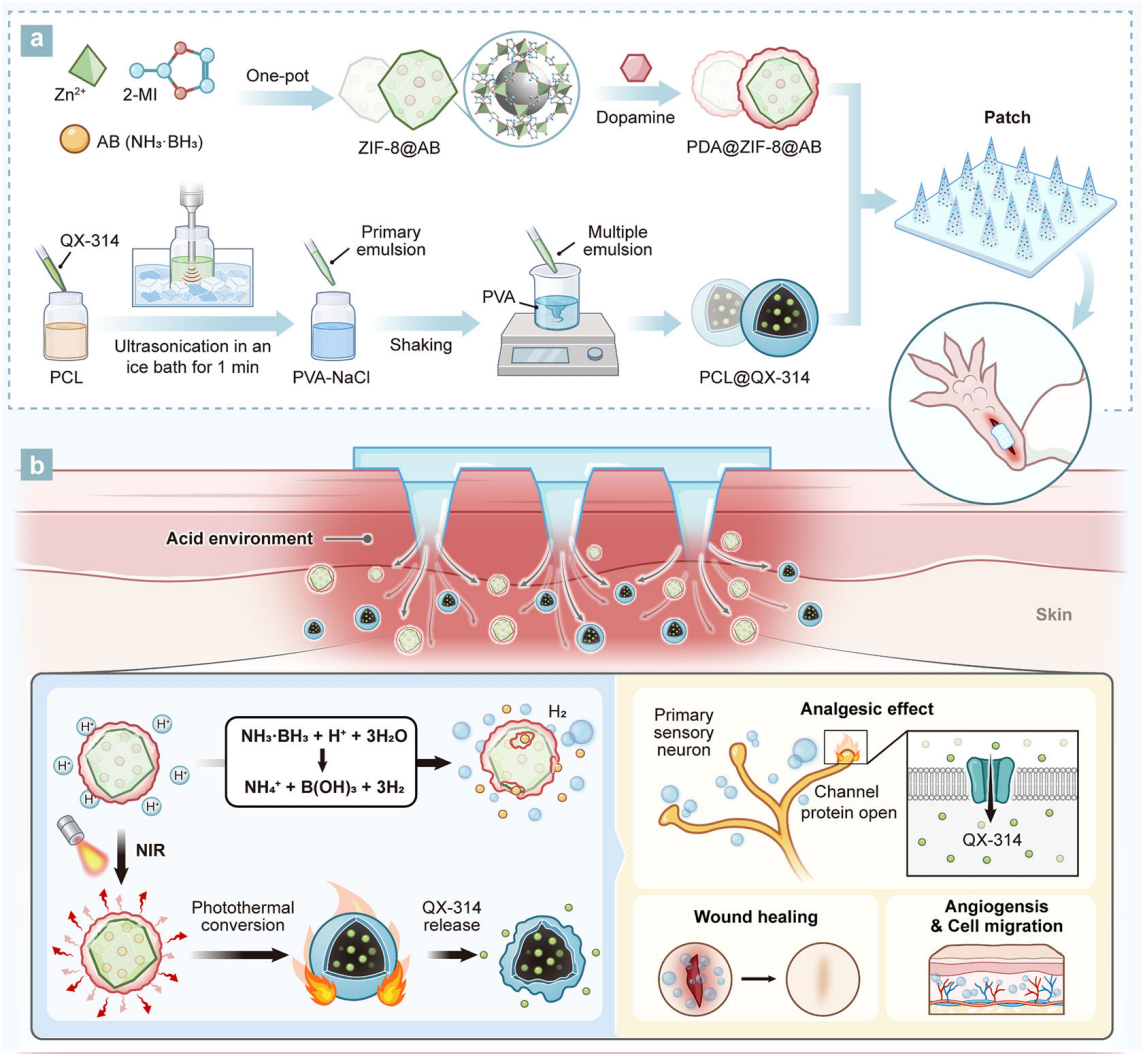
Schematic illustration of the H_2_‐releasing and photothermal‐triggered MN patch fabrication and its therapeutic mechanism. a) Synthesis and preparation process of PDA@ZIF‐8@AB NPs and PCL@QX‐314 MSs. ZIF‐8@AB was synthesized via a one‐pot reaction, with AB encapsulated for acid‐responsive H_2_ release, then coated with PDA and incorporated into the MN patch. PCL@QX‐314 MSs were prepared by emulsion and integrated into the MN system. b) Mechanism of action in the postoperative incision environment. In the acidic wound environment, PDA@ZIF‐8@AB NPs release H_2_ that reduces oxidative stress and inflammation, providing sustained analgesic effects while promoting angiogenesis and cell migration. NIR irradiation activates PDA, inducing a photothermal effect that triggers controlled QX‐314 release and opens membrane ion channels, enhancing localized pain relief. NPs: nanoparticles; PCL@QX‐314 MSs: PCL microspheres loaded with QX‐314.

To tackle these issues, we propose a novel therapeutic strategy: a transdermal drug delivery system that utilizes molecular hydrogen delivered via a photothermal MN. This approach enables localized H_2_ delivery to control postoperative incisional pain and tissue healing, while also allowing for the on‐demand release of the local analgesic (QX‐314) tailored to individual pain thresholds (**Figure** **1**), optimizing postoperative incision management. The system utilizes MNs as a drug delivery vehicle, which have emerged as a promising transdermal delivery system.^[^ ^15^ ^]^ There are three steps in the development of this H_2_‐based therapeutic system.

The first step in this analgesic delivery system involves using ammonia borane (AB) as an H_2_ donor,^[^ ^16^ ^]^ as it is an efficient hydrogen storage material that exhibits acid‐responsive H_2_ release behavior, ensuring a stable supply of H_2_ in the acidic microenvironment of postoperative wounds.^[^ ^17^ ^]^ First of all, AB is encapsulated within ZIF‐8 using a one‐step synthesis method, resulting in composite ZIF‐8@AB nanoparticles (NPs). Next, this composite undergoes a self‐polymerization reaction with dopamine hydrochloride to generate PDA@ZIF‐8@AB (Figure 1a). The acid‐sensitive degradation of PDA@ZIF‐8@AB ensures the gradual release of H_2_, providing localized analgesic and therapeutic effects around the incision site. This H_2_‐release provides effective pain relief up to five days while promoting tissue regeneration, thereby facilitating a smoother healing process (Figure 1b).

The second step addresses the inherent subjectivity of pain, which is influenced by factors such as genetics, psychological state, and prior pain experiences.^[^ ^18^ ^]^ To address the current “one‐size‐fits‐all” approach in clinical postoperative pain management,^[^ ^19^ ^]^ we incorporated a personalized pain management strategy into the system. In the current study, QX‐314, a quaternary ammonium derivative of lidocaine, which can enter primary sensory neurons through activated membrane ion channels, resulting in targeted regional anesthesia,^[^ ^1^, ^20^ ^]^ is applied to provide supplementary on‐demand analgesia. QX‐314‐loaded polycaprolactone (PCL) microspheres (MSs) are prepared using a water/oil/water (W/O/W) emulsion‐solvent evaporation method (Figure 1a). Under near‐infrared (NIR) laser irradiation, the photothermal conversion of PDA triggers the deformation of PCL MSs, enabling the on‐demand release of QX‐314. The activation of ion channels at elevated temperatures promotes the entry of QX‐314 into neurons, providing targeted pain relief (Figure 1b). This mechanism allows for personalized treatment tailored to individual patient pain sensitivity, effectively bridging the gap between standardized pain management and personalized care.

The final step involves delivering the above‐mentioned formulations (PDA@ZIF‐8@AB NPs and PCL@QX‐314 MSs) to the surgical incision site using hyaluronic acid (HA)‐based dissolvable MNs (Figure 1b). We validated the effectiveness of this system using an animal model with plantar incisions and investigated the analgesic and promoting wound healing mechanisms of H_2_ through RNA sequencing (RNA‐seq) techniques.

This system integrates innovative analgesic strategies and regenerative therapies to tackle the dual challenges of postoperative pain relief and wound healing, in line with the trend of Enhanced Recovery After Surgery (ERAS).

## 2 Results

### 2.1 Optimization and Characterization of PDA@ZIF‐8@AB NPs

**Figure 2:**
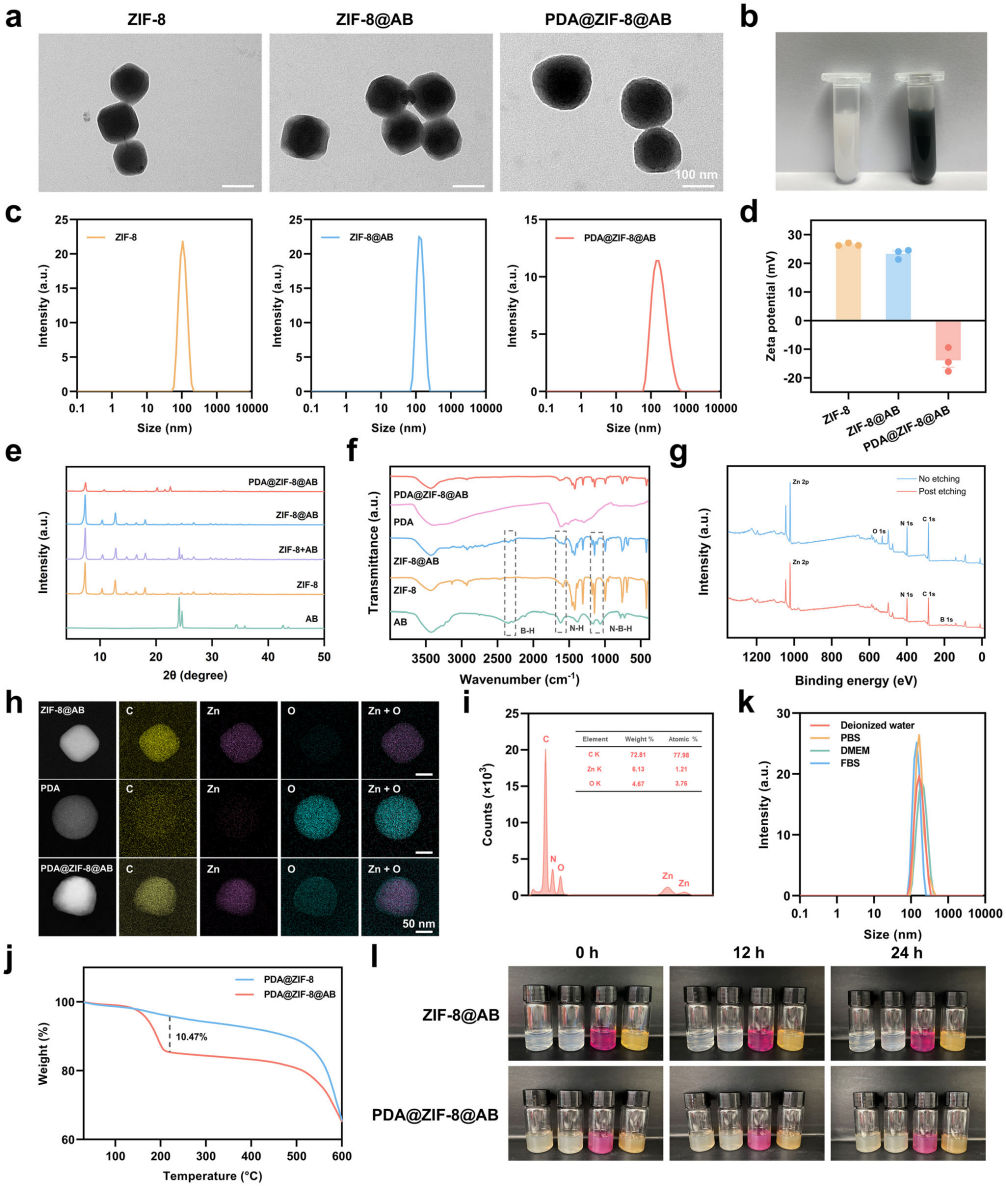
Characterization of PDA@ZIF‐8@AB NPs. a) TEM images of ZIF‐8, ZIF‐8@AB, and PDA@ZIF‐8@AB NPs. b) Visual appearance of ZIF‐8@AB and PDA@ZIF‐8@AB NPs dispersions. c) Particle size distribution of NPs as determined by DLS. d) Zeta potential measurements for NPs. e‐f) XRD patterns (e) and FT‐IR spectra (f) of different materials. g XPS spectra before and after etching for PDA@ZIF‐8@AB NPs. h) EDS mapping images of ZIF‐8@AB, PDA, PDA@ZIF‐8@AB NPs. i) EDS quantification of elemental composition in PDA@ZIF‐8@AB NPs. j) TGA curves of PDA@ZIF‐8 and PDA@ZIF‐8@AB NPs. k) DLS measurements of PDA@ZIF‐8@AB NPs in various media over 24 h. l) Photographic images of ZIF‐8@AB and PDA@ZIF‐8@AB NPs in different media at 0, 12, and 24 h.

We optimized the synthesis of PDA@ZIF‐8@AB NPs by assessing several critical parameters, including the reaction medium, the method of AB addition, the stirring duration for the preparation of ZIF‐8@AB, the concentration of dopamine hydrochloride (DA), and the reaction time for DA self‐polymerization and modification. This comprehensive analysis enabled us to establish the optimal preparation protocol for the NPs (Figure S1, Supporting Information). Following PDA modification, the nanomaterial transitioned from a milky white to a deep gray color (**Figure** **2**b). The microstructural characteristics of ZIF‐8, ZIF‐8@AB, and PDA@ZIF‐8@AB were examined using transmission electron microscopy (TEM). Both ZIF‐8 and ZIF‐8@AB exhibited uniform rhombic dodecahedral morphologies, consistent with previously reported structures.^[^ ^21^ ^]^ In contrast, PDA@ZIF‐8@AB displayed a morphology that was closer to spherical, featuring a rough surface with a granular texture (Figure 2a).^[^ ^22^ ^]^ The particle sizes were measured at 103 ± 3 nm for ZIF‐8, 135 ± 6 nm for ZIF‐8@AB, and 177 ± 5 nm for PDA@ZIF‐8@AB (Figure 2c), indicating that the modifications led to a significant increase in particle size, likely due to the incorporation of PDA and AB into the structure. Following PDA modification, the surface charge of the NPs shifted from positive to negative, significantly enhancing their retention in biological tissues and thereby improving therapeutic efficacy (Figure 2d).^[^ ^23^ ^]^

Further investigation of the crystalline structure of the NPs was conducted using X‐ray diffraction (XRD). The results indicated no significant changes in the position and intensity of the diffraction peaks for ZIF‐8@AB compared to blank ZIF‐8; however, notable alterations were observed in PDA@ZIF‐8@AB. These findings supported the TEM results, suggesting that the loading of AB did not disrupt the crystalline structure of ZIF‐8, while the surface polymerization of PDA did have an effect (Figure 2e). Fourier transform infrared spectroscopy (FT‐IR) confirmed the structured composition of the NPs. The distinct N‐B‐H stretching vibration peak at ≈1100 cm⁻^1^ and the B‐H stretch around 2500 cm⁻^1^ of AB in the ZIF‐8@AB spectrum confirmed the successful encapsulation of AB within the ZIF‐8 framework. Additionally, the N‐H peak between 1500 and 1600 cm⁻^1^ in the ZIF‐8@AB spectrum indicated the successful incorporation of AB and its interaction with ZIF‐8. No significant shifts were observed in the ZIF‐8 and ZIF‐8@AB spectra, indicating that the crystalline structure of ZIF‐8 remained intact post‐loading. After PDA modification, the slight broadening and shifts in peaks, especially between 1400 and 1600 cm⁻^1^, indicated successful surface polymerization of PDA, introducing new functional groups without affecting the ZIF‐8 structure (Figure 2f). In the ultraviolet‐visible (UV‐vis) absorption spectrum, a new absorption peak between 280 and 290 nm was observed in the PDA@ZIF‐8@AB sample, which was absent in the ZIF‐8@AB spectrum. This peak was attributed to the π‐π* transition of the catechol groups present in dopamine, confirming the successful modification with PDA (Figure S2c, Supporting Information).

X‐ray photoelectron spectroscopy (XPS) was conducted to confirm the surface composition and structural modifications of NPs. Before etching, characteristic peaks corresponding to zinc (Zn 2p), nitrogen (N 1s), carbon (C 1s), and oxygen (O 1s) were observed, indicating the presence of the ZIF‐8 structure and successful PDA surface modification. The prominent O 1s signal confirmed effective PDA coating. After etching, the oxygen signal decreased significantly, while the Zn 2p peak became more pronounced, indicating the removal of the PDA layer and the exposure of the ZIF‐8 core. The appearance of a boron (B 1s) peak post‐etching provided evidence for the encapsulation of AB within the ZIF‐8 framework (Figure 2g). Additional confirmation was obtained through TEM mapping (Figure 2h) and energy‐dispersive X‐ray spectroscopy (EDS) analysis (Figure 2i), which visualized the distribution of carbon, zinc, and oxygen, further affirming the structure of the NPs. Based on thermogravimetric analysis (TGA), the drug loading of AB in the PDA@ZIF‐8@AB NPs was calculated to be ≈10% (Figure 2j), with an encapsulation efficiency of around 17% (Figure S2a, Supporting Information).

### 2.2 Stable Dispersion and Enhanced Biocompatibility after PDA Modification

To evaluate the impact of PDA modification on the stability of NPs, ZIF‐8@AB and PDA@ZIF‐8@AB NPs were placed in equal volumes of various solutions, including deionized water, phosphate‐buffered saline (PBS), Dulbecco's Modified Eagle Medium (DMEM), and fetal bovine serum (FBS), at the same concentration and allowed to stand. As shown in Figure 2l, both types of particles remained uniformly dispersed in all four solutions over a 24‐hour period, with no visible signs of sedimentation. Dynamic light scattering (DLS) measurements indicated that the particle size of ZIF‐8@AB (Figure S2b, Supporting Information) and PDA@ZIF‐8@AB NPs (Figure 2k) in each solution did not exhibit significant changes during this time. The consistent particle size across different media demonstrated that the PDA modification did not compromise the stability of the NPs. This stable dispersion, without aggregation, suggested that the modified NPs could maintain uniformity in diverse biological environments, which was beneficial for practical applications.

**Figure 3:**
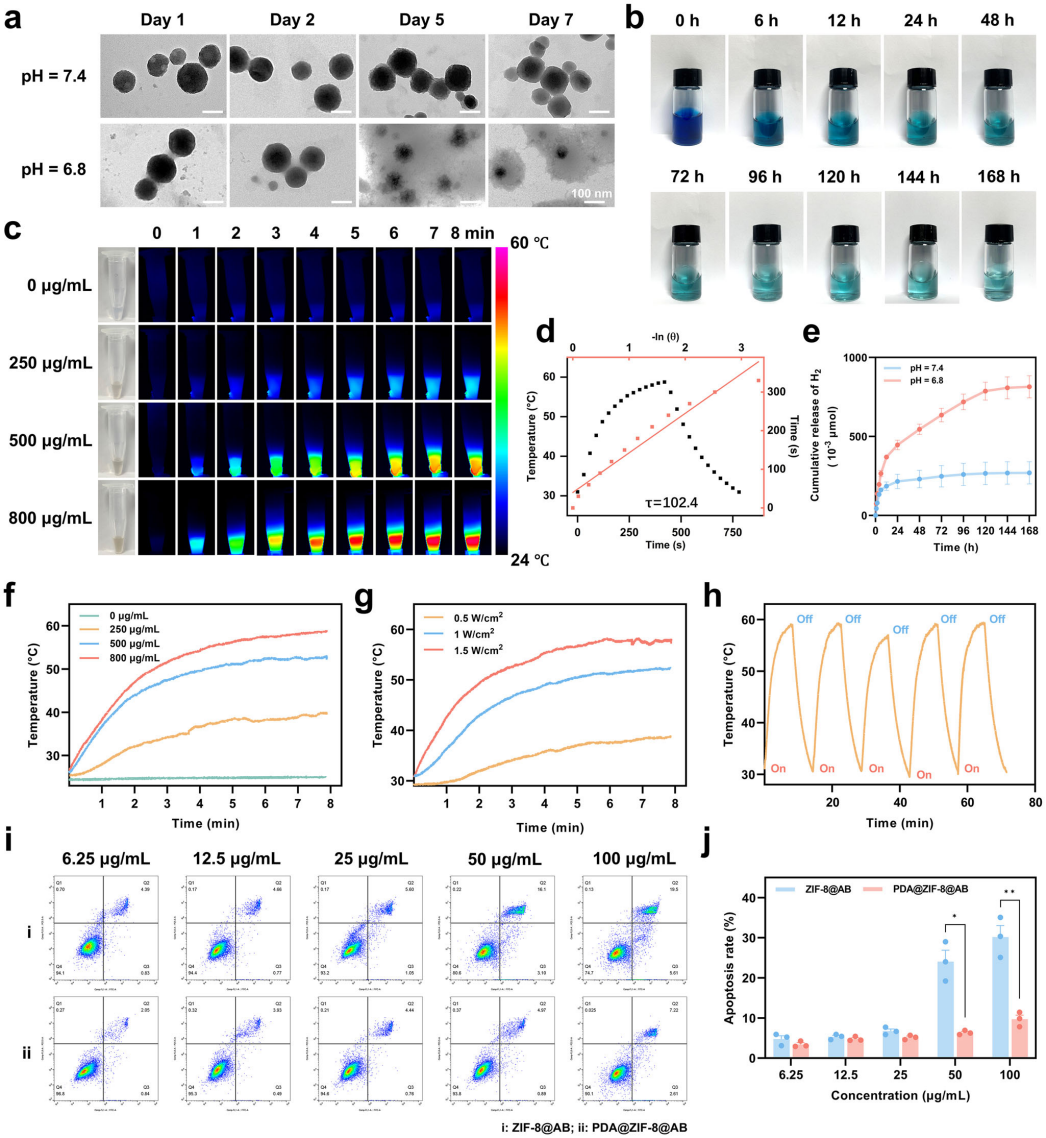
Acid‐responsive H_2_ release, photothermal properties, and cytotoxicity of PDA@ZIF‐8@AB NPs. a) TEM images of PDA@ZIF‐8@AB NPs at different time points under pH 7.4 and pH 6.8 conditions. b) Photographic images of PDA@ZIF‐8@AB NPs in MB‐Pt probe solution at various time intervals over 168 h. c) Thermal images of PDA@ZIF‐8@AB dispersions at different concentrations under NIR irradiation (1.0 W cm^−2^) for 8 min. d) Photothermal conversion efficiency calculation for PDA@ZIF‐8@AB NPs. e) In vitro cumulative H_2_ release profiles of PDA@ZIF‐8@AB NPs at pH 7.4 and pH 6.8. f‐g) Temperature elevation of PDA@ZIF‐8@AB NPs at various concentrations (1.0 W cm^−2^) and NIR power densities (500 µg mL^−1^) over 8 min. h) Temperature cycling of PDA@ZIF‐8@AB NPs (500 µg mL^−1^) under repeated NIR irradiation (1.5 W cm^−2^) on‐off cycles. i) Flow cytometry analysis of apoptosis rates in cells treated with varying concentrations of ZIF‐8@AB and PDA@ZIF‐8@AB NPs. j) Quantification of apoptosis rates, comparing ZIF‐8@AB and PDA@ZIF‐8@AB NPs treatments at different concentrations. Data represented mean ± SEM from *n* = 3 independent experiments. *P* value: ^*^ *p* < 0.05 and ^**^ *p* < 0.01.

ZIF‐8 exhibits dose‐dependent biocompatibility. One study demonstrated that at concentrations below 30 µg mL^−1^, ZIF‐8 did not exhibit significant cytotoxicity. However, at higher concentrations, the release of Zn^2^⁺ ions induced mitochondrial reactive oxygen species (ROS) production, causing irreversible DNA damage and activating apoptotic pathways.^[^ ^17a^ ^]^ In our research, we aimed to explore whether PDA modification could improve the biocompatibility of ZIF‐8 NPs. The apoptosis rate of cells treated with ZIF‐8@AB and PDA@ZIF‐8@AB NPs was evaluated using an Annexin V‐FITC/PI apoptosis kit. As shown in **Figure** **3**i, the apoptosis rate increased with the concentration of NPs; however, it was lower in the PDA@ZIF‐8@AB group compared to the ZIF‐8@AB group across all concentrations. This finding indicated that PDA modification significantly improved the biocompatibility of the NPs. At the highest concentration (100 µg mL^−1^), the apoptosis rate for ZIF‐8@AB reached 30 ± 2%, whereas it was only 9.7 ± 1.0% for PDA@ZIF‐8@AB (Figure 3j). These results suggested that PDA coating reduced the cytotoxicity of ZIF‐8@AB NPs, thereby enhancing their potential for safe biomedical applications.

### 2.3 Acid‐Triggered Degradation and Sustained H_2_ Release from PDA@ZIF‐8@AB NPs

The acid‐triggered degradation of PDA@ZIF‐8@AB NPs was evaluated under different pH conditions (pH 7.4 and pH 6.8). TEM analysis revealed distinct degradation behaviors depending on the pH environment (Figure 3a). At pH 7.4, the NPs largely maintained their structural integrity throughout the 7‐day observation period, with only minor morphological changes observed. In contrast, at pH 6.8, degradation was notably accelerated. By day 5, the particles exhibited significant structural disintegration, with their shapes becoming progressively indistinct and blurred. By day 7, the NPs had almost entirely disintegrated, displaying clear signs of breakdown and degradation. These results underscored the acid‐sensitive nature of the PDA@ZIF‐8@AB NPs, suggesting their potential for targeted drug release in low‐pH environments, such as wound sites or areas of inflammation.

H_2_ was detected using the MB‐Pt probe,^[^ ^24^ ^]^ clearly demonstrating the crucial role of AB in the NP system's ability to generate H_2_. As illustrated in the series of photographs, under acidic conditions (pH 6.8), the color of the MB‐Pt probe solution containing PDA@ZIF‐8@AB NPs gradually faded over time (Figure 3b), while the solution with PDA@ZIF‐8 NPs exhibited almost no fading during the same period (Figure S3c, Supporting Information). These observations suggested that AB was essential for the release of H_2_ in the PDA@ZIF‐8@AB formulation.

The cumulative release curves further demonstrated that under acidic conditions (pH 6.8), PDA@ZIF‐8@AB NPs continuously released H_2_ over a period of 120 hours, with the release gradually plateauing between 120 and 168 hours. In contrast, at pH 7.4, the release rate was slower (Figure 3e). Additionally, a comparison with the release curves of free AB indicated that encapsulating AB within the NPs facilitated for a more gradual and sustained release, highlighting the advantages of this system for applications requiring prolonged H_2_ delivery (Figure S3b, Supporting Information). These findings underscored the efficiency of PDA@ZIF‐8@AB NPs in achieving controlled, pH‐sensitive H_2_ release, suggesting significant potential for therapeutic applications.

### 2.4 Excellent Photothermal Properties of PDA@ZIF‐8@AB NPs

The modification of PDA not only reduced the toxicity of ZIF‐8 and enhanced its stability but also endowed the NPs with photothermal conversion capabilities, allowing for controlled drug release.^[^ ^25^ ^]^ The photothermal properties of PDA@ZIF‐8@AB NPs were evaluated under 808 nm NIR laser irradiation, and the results demonstrated excellent photothermal conversion performance. As shown in the infrared thermal images and temperature variation curves (Figure 3c,f,g), the temperature increased with rising concentrations of the materials (0, 250, 500, 800 µg mL^−1^) and NIR power densities (0.5, 1.0, 1.5 W cm^−2^), following a nonlinear upward trend until reaching a plateau.

At a laser power density of 1.0 W cm^−2^, the 800 µg mL^−1^ PDA@ZIF‐8@AB suspension reached a temperature of 59 °C within 8 min. In comparison, suspensions with concentrations of 500 µg mL^−1^ and 250 µg mL^−1^ reached peak temperatures of 53 °C and 40 °C, respectively. The control group (0 µg mL^−1^) exhibited negligible temperature changes under the same irradiation conditions (Figure 3f). When exposed to a power density of 1.5 W cm^−2^, the 500 µg mL^−1^ suspension exceeded 58 °C during laser irradiation, while the temperature increase was more moderate at 1.0 W cm^−2^ and 0.5 W cm^−2^, reaching ≈52 °C and 41 °C, respectively. Notably, PDA@ZIF‐8@AB NPs displayed excellent photothermal stability during cyclic NIR irradiation (Figure 3h), with consistent temperature elevations observed over five “on/off” cycles. The calculated photothermal conversion efficiency (η) for these NPs was 41% (Figure 3d), highlighting their potential as effective agents for photothermal therapy.

### 2.5 Preparation and Characterization of PCL@QX‐314 MSs

**Figure 4:**
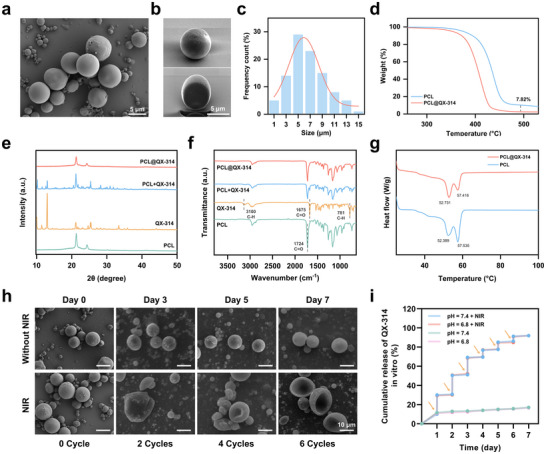
Characterization, thermal stability, and release profile of PCL@QX‐314 MSs. a) SEM image showing the surface morphology of PCL@QX‐314 MSs. b) High‐magnification SEM images of individual PCL@QX‐314 MS. c) Size distribution histogram of PCL@QX‐314 MSs. d) TGA curves of PCL and PCL@QX‐314 MSs. e‐f) XRD patterns (e) and FT‐IR spectra (f) of different particles. g) DSC curves of PCL and PCL@QX‐314 MSs. h) SEM images showing the stability of PCL@QX‐314 MSs with multiple NIR irradiation cycles and without irradiation over 7 days. i) In vitro cumulative release profiles of QX‐314 from PCL@QX‐314 MSs under different pH conditions with and without NIR irradiation over 7 days. Arrows in the graph indicated the time points of NIR irradiation. Data represented mean ± SEM from *n* = 3 independent experiments.

PCL is a biodegradable and biocompatible polymer widely recognized for its excellent properties in biomedical applications, including low toxicity, mechanical flexibility, and superior thermal performance.^[^ ^26^ ^]^ These characteristics make it particularly suitable for controlled‐release systems. Moreover, its ability to undergo temperature‐induced shape changes further enhances its potential in drug‐controlled release delivery systems.^[^ ^27^ ^]^ In this study, PCL and QX‐314 were selected as the core materials for the preparation of MSs following established methods,^[^ ^28^ ^]^ and the stabilizer in the inner aqueous phase was optimized to improve the quality of the MSs. Scanning electron microscopy (SEM) images (Figure S4a–c, Supporting Information) revealed significant differences in surface morphology and size distribution based on the stabilizer used (Blank, PEG, and HA). MSs without stabilizers (Blank) exhibited irregular shapes and rough surfaces. In contrast, PEG‐stabilized MSs had smoother surfaces but larger particle sizes, which may limit their application in MN‐based drug delivery systems. However, HA‐stabilized MSs (**Figure** **4**a,b) demonstrated higher density, smaller, and more uniform particle sizes, indicating their suitability for drug delivery applications. Cross‐sectional images obtained using ion‐slicing technology demonstrated a dense internal structure, suggesting enhanced mechanical stability (Figure 4b).^[^ ^29^ ^]^ This compact arrangement reflected a robust MS formation process, which could improve the overall durability of the MSs during handling and delivery. Particle size distribution analysis indicated that the majority of the MSs ranged between 3 and 10 µm, with an average diameter of 6.6 ± 1.5 µm (Figure 4c).

The XRD analysis demonstrated the crystalline characteristics of both PCL and QX‐314 (Figure 4e). In the physical mixture of PCL and QX‐314, both sets of peaks were observed, indicating no significant structural changes in either component. However, in the PCL@QX‐314 MSs, the reduction in the intensity of the QX‐314 peaks suggested that the drug's crystalline structure had been partially masked during encapsulation, indicating successful dispersion and encapsulation of QX‐314 within the PCL matrix. The FT‐IR spectrum (Figure 4f) provided deeper insights into the chemical interactions within the MSs. Key characteristic peaks of PCL, such as the C═O stretching vibration at 1724 cm⁻^1^, were clearly observed. For QX‐314, the spectrum revealed characteristic peaks between 1630 and 1680 cm⁻^1^ (C═O stretching) and 3000–3100 cm⁻^1^ (aromatic C─H stretching), confirming its chemical structure. In the encapsulated PCL@QX‐314 MSs, shifts and reductions in the intensity of these peaks, particularly in the C═O stretching region, suggested molecular interactions between PCL and QX‐314 within the MSs. These spectral changes confirmed successful drug encapsulation and provided evidence of the chemical interaction between the polymer and the encapsulated drug.

TGA was employed to evaluate the thermal stability and drug loading efficiency of the MSs. The TGA curve (Figure 4d) showed a characteristic weight loss profile for PCL@QX‐314, featuring two primary stages of degradation. The initial weight loss corresponded to the degradation of the encapsulated drug, while the second stage was associated with the degradation of the PCL matrix. Based on the TGA results, the drug loading of the PCL@QX‐314 MSs was calculated to be ≈8%, with an encapsulation efficiency of around 45% (Figure S4d, Supporting Information). These values confirmed the successful incorporation of QX‐314 into the PCL matrix. Differential Scanning Calorimetry (DSC) was employed to further evaluate the thermal properties of the PCL@QX‐314 MSs (Figure 4g). The melting point of PCL was observed at around 57.5 °C, which aligned with the established thermal properties of PCL.^[^ ^30^ ^]^ In the PCL@QX‐314 MSs, minor shifts in the thermal transition peaks were detected, indicating that the encapsulated drug influenced the thermal behavior of the polymer. Based on these DSC results and additional thermal testing data, a laser irradiation condition of 1.0 W cm^−2^ for 5 min was selected for the controlled release of QX‐314. The temperature rise induced by this irradiation also facilitated the opening of TRPV1 channels, allowing QX‐314 to enter cells and exert its analgesic effect.

### 2.6 Thermally Induced Deformation and Photothermal‐Controlled Release of PCL@QX‐314 MSs

The thermally induced deformation and photothermal‐controlled release of PCL@QX‐314 MSs were systematically investigated by co‐incubating the MSs with PDA@ZIF‐8@AB NPs. Since PCL lacks inherent photothermal conversion capabilities, the heating effect under NIR irradiation was primarily driven by the PDA component, which efficiently absorbed NIR energy and generated localized heat. Morphological analysis using SEM (Figure 4h) revealed that, in the absence of NIR irradiation, the MSs maintained their structural integrity with smooth, spherical surfaces over a 7‐day period. However, upon exposure to NIR irradiation (1.0 W cm^−2^, 5 min), significant structural changes occurred in the MSs after multiple irradiation cycles. By the fourth to sixth cycles, the MSs exhibited clear deformation, characterized by surface irregularities and partial collapse. These results suggested that the photothermal effect induced by NIR irradiation caused softening and deformation of the PCL matrix, thereby facilitating the release of the encapsulated QX‐314.

The release of QX‐314 from PCL@QX‐314 MSs was evaluated under various pH conditions (Figure 4i). In the absence of NIR irradiation, the release of QX‐314 remained minimal (below 20%) at both pH 7.4 and 6.8 over a period of 7 days, indicating the strong stability of the MSs without external stimuli. However, under NIR irradiation, a significant stepwise increase in release was observed with each irradiation cycle, resulting in over 90% of QX‐314 being released after six cycles in both pH environments. These results suggested that pH had a little effect on the release, with the primary trigger being the NIR‐induced photothermal effect. The stepwise release pattern highlighted the effectiveness of NIR as a precise trigger for controlled drug release, demonstrating the potential of PCL@QX‐314 MSs for on‐demand drug delivery.

### 2.7 Morphology, Mechanical Properties, and Skin Penetration Efficiency of PDA@ZIF‐8@AB and PCL@QX‐314‐Loaded MNs

**Figure 5:**
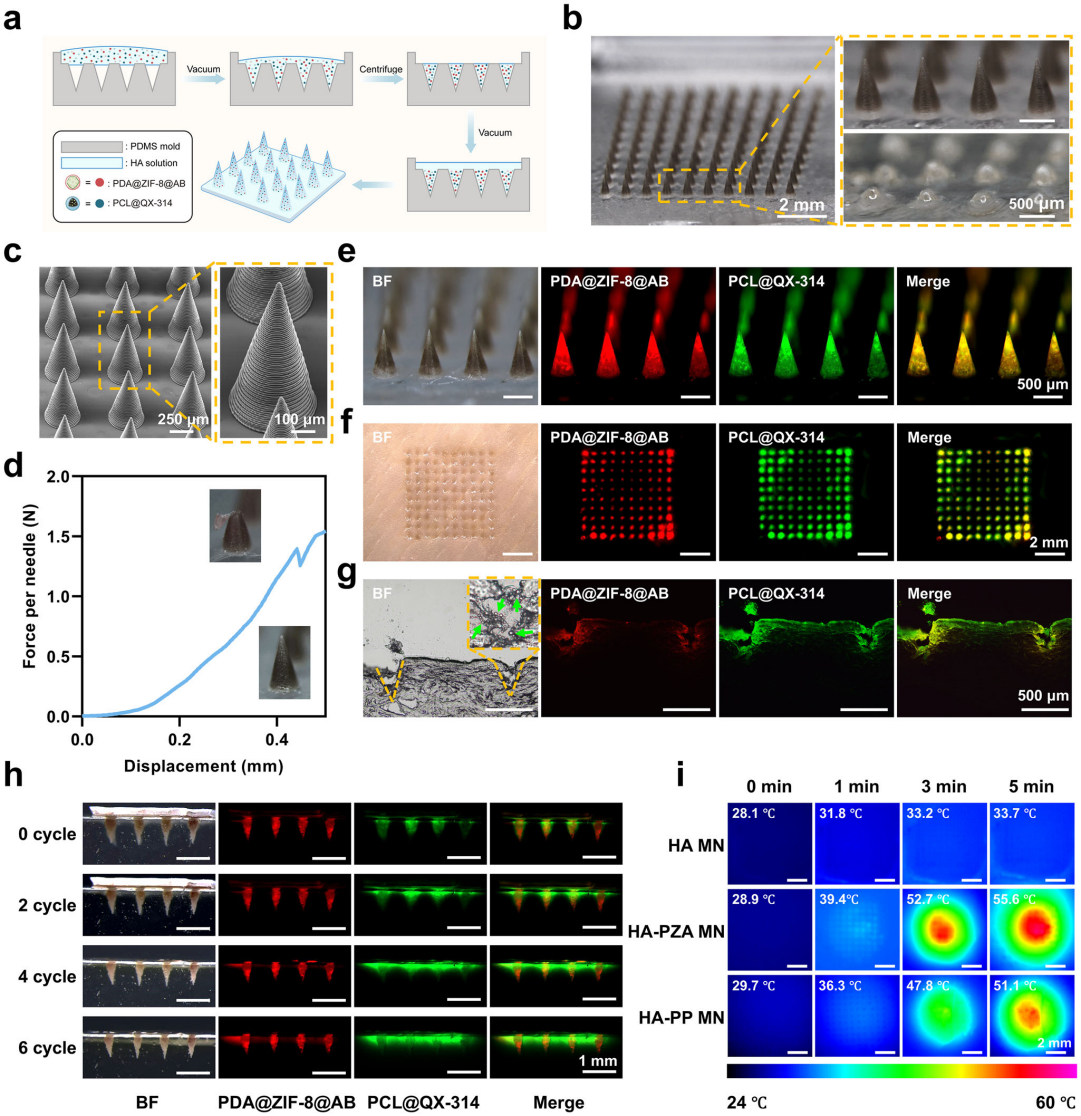
Preparation, characterization, mechanical properties, and photothermal properties of HA‐PP MN patch. a) Schematic illustration of the fabrication process of the HA‐PP MN patch. b) Optical images of the fabricated HA‐PP MN patch, with an enlarged view showing the MN array. c) SEM images of the HA‐PP MN patch. d) Force‐displacement curve of the HA‐PP MN patch (Inset: optical images of MNs before and after the mechanical strength test). e) Fluorescence imaging of the HA‐PP MN patch showing the distribution of PDA@ZIF‐8@AB NPs and PCL@QX‐314 MSs within the MNs. f) Optical images of the HA‐PP MN patch applied to porcine skin. g) Images of frozen sections of MNs inserted in porcine skin. The arrows in the images indicated the PCL@QX‐314 MSs loaded within the MNs, which successfully remained in the skin after MN insertion. h) Stability test of the HA‐PP MN patch under multiple cycles of NIR irradiation (1 W cm^−2^). i) Infrared thermographic images of different MN patches under NIR irradiation (1 W cm^−2^), illustrating temperature elevation over time. HA‐PP MN patch: the HA‐based MN patch loaded with PDA@ZIF‐8@AB NPs and PCL@QX‐314 MSs.

PDA@ZIF‐8@AB NPs and PCL@QX‐314 MSs were encapsulated in HA‐based dissolvable MN patches (HA‐PP MN) using a layer‐by‐layer casting technique (**Figure** **5**a).^[^ ^31^ ^]^ Optical images revealed that the HA‐PP MNs formed a well‐organized 10 × 10 array on a 7 mm × 7 mm patch, featuring sharp and well‐defined needle tips (Figure 5b). The incorporation of PDA@ZIF‐8@AB NPs imparted a dark coloration to the tips of the needles, which were anticipated to facilitate precise drug delivery into the skin upon application (Figure 5b). Each needle exhibited a base diameter of 400 µm and a height of 850 µm. High‐magnification SEM images confirmed the structural integrity of the needles, displaying uniform conical shapes and well‐preserved surface morphology (Figure 5c).

The drug loading capacity of the patches was assessed, revealing that each patch contained ≈285 ± 9 µg of PDA@ZIF‐8@AB NPs and 241 ± 7 µg of PCL@QX‐314 MSs, indicating successful loading of both components (Figure S5a, Supporting Information). Mechanical strength testing (Figure 5d) demonstrated that each needle could withstand forces of up to 1.5 ± 0.5 N before bending or breaking, significantly exceeding the 0.1 N required for effective skin penetration.^[^ ^32^ ^]^ This confirmed that the MN patches possessed sufficient mechanical robustness for transdermal application without compromising their structural integrity.

Fluorescence visualization was performed using Rhodamine B and fluorescein sodium as model drugs for NPs and MSs, respectively (Figure 5e). Following application to porcine skin, the MNs achieved over 99% drug delivery, successfully penetrating the skin (Figure 5f). Analysis of the frozen sections revealed that Rhodamine B remained well encapsulated within the NPs, while fluorescein sodium exhibited slight diffusion due to the inherent burst release characteristics of water‐soluble drugs in MSs (Figure 5g). The overlay of fluorescence signals confirmed the successful co‐delivery of both components into the skin tissue. These findings highlighted the effective design and mechanical stability of the HA‐PP MN patches, enabling efficient transdermal delivery of therapeutic agents.

### 2.8 Photothermal Activation and Visualization of Laser‐Controlled Drug Release in HA‐PP MN Patches

To investigate the photothermal response and controlled drug release of HA‐based MN patches, we prepared three groups of patches: blank HA MN, HA‐PZA MN containing only PDA@ZIF‐8@AB (PZA) NPs, and HA‐PP MN. The objective was to determine whether the PDA component in the patches could generate sufficient heat under NIR irradiation to facilitate targeted drug release. Upon exposure to an 808 nm NIR laser (1.0 W cm^−2^), thermal imaging results showed minimal temperature changes in the blank HA MN patches. In contrast, both HA‐PZA MN and HA‐PP MN patches exhibited rapid temperature increases, with the HA‐PP MN group reaching 51 °C after 5 min, thereby meeting the experimental requirements for triggering the controlled release of QX‐314 from the PCL@QX‐314 MSs (Figure 5i). This heating effect confirmed the role of PDA@ZIF‐8@AB NPs in converting NIR energy into localized heat within the MN patches.

To evaluate laser‐controlled drug release, HA‐PP MN patches were inserted into a skin‐mimicking agarose gel and subjected to repeated NIR irradiation cycles (Figure 5h). Rhodamine B, encapsulated in NPs as a stable model drug, exhibited minimal diffusion into the gel throughout the cycles, likely due to the structural stability of the NPs under heat. This limited diffusion indicated that repeated NIR stimulation did not significantly affect the drug release rate from the NPs. In contrast, fluorescein sodium, encapsulated within the PCL MSs, demonstrated a different behavior. Designed to respond to the photothermal effect, fluorescein sodium exhibited a stepwise increase in release with each NIR cycle. The heat generated by PDA in the MN patches effectively triggered the diffusion of fluorescein sodium, resulting in an intensified and prolonged fluorescence signal in the gel with successive irradiations. This controlled, on‐demand release pattern highlighted the potential of HA‐PP MN patches for applications requiring precise, NIR‐triggered drug delivery.

### 2.9 PDA@ZIF‐8@AB NPs and PCL@QX‐314 MSs Retention and Drug Release in Local Tissue Microenvironment

To evaluate the retention and sustained release of PDA@ZIF‐8@AB NPs and PCL@QX‐314 MSs in the local tissue microenvironment, fluorescent HA‐PP MN patches were applied for in vivo rat paw skin puncture testing.

Fluorescence images were captured daily to monitor the retention and release of NPs and MSs. The results showed that after the application of HA‐PP MN patches, the NPs and MSs were effectively delivered to the tissue and remained stably localized at the application site. PDA@ZIF‐8@AB NPs primarily relied on an acid‐responsive mechanism for the gradual release of the fluorescent model drug, with fluorescence remaining detectable for up to 5 days. The presence or absence of NIR irradiation had minimal impact on their release and degradation (Figure S6a, b, Supporting Information). In contrast, PCL@QX‐314 MSs showed significant changes under NIR irradiation. Without NIR exposure, PCL@QX‐314 MSs remained localized in the tissue with minimal drug release (Figure S6a, Supporting Information). However, with daily NIR exposure, the fluorescence intensity gradually decreased with each light cycle, and by Day 5, the fluorescence had nearly disappeared (Figure S6b, Supporting Information).

### 2.10 Enhanced Cell Migration and Wound Healing Driven by the H_2_‐Releasing of MN Patches

**Figure 6:**
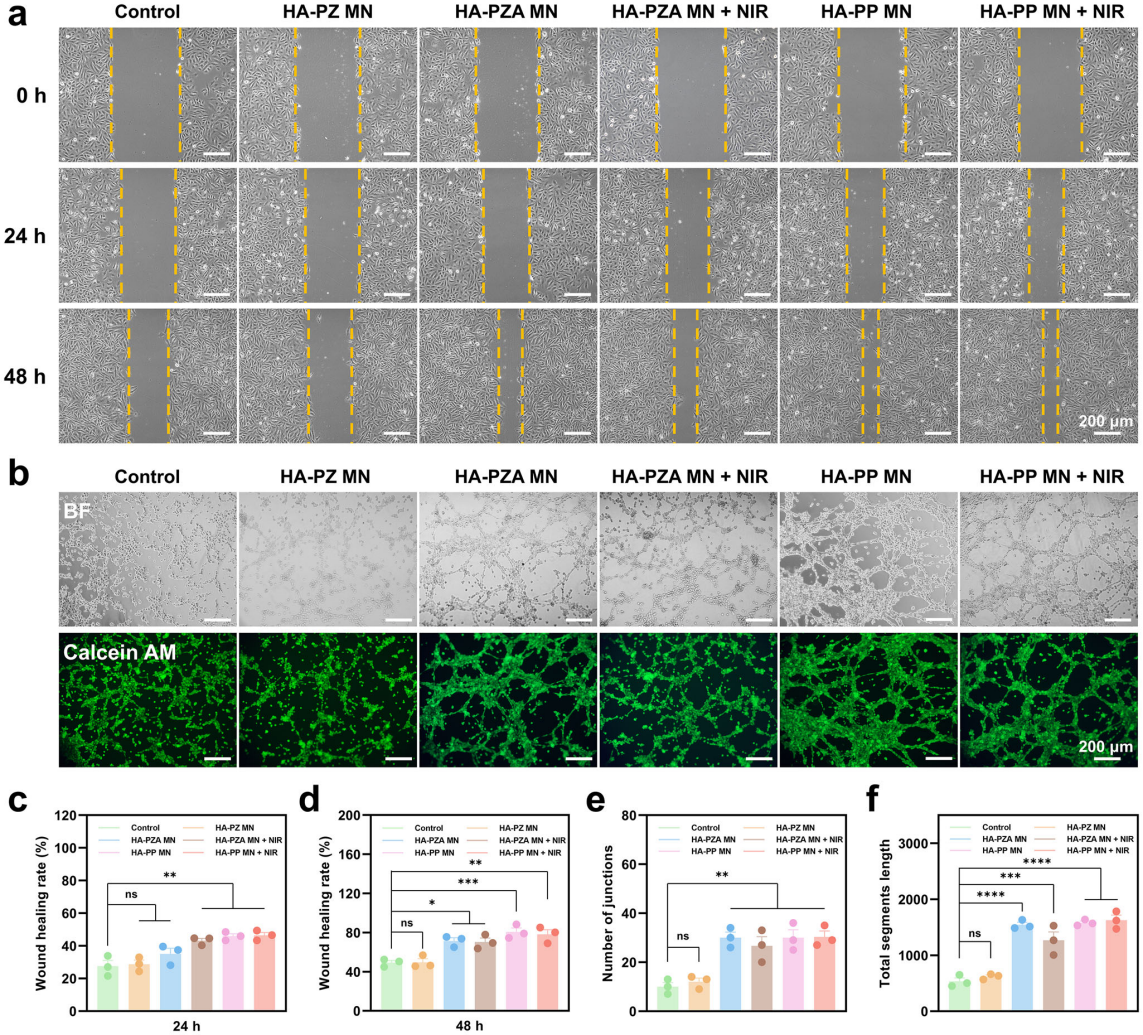
Evaluation of wound healing and angiogenic effects of various MN patches on HUVEC cells. a) Bright field images showing the scratch assay of HUVEC treated with different MN patches at 0, 24, and 48 h. b) Bright field and Calcein‐AM staining images of HUVEC cells after treatment with different MN patches, demonstrating tube formation and angiogenic network structures. c) Quantification of wound healing rate at 24 h for each treatment group. d) Quantification of wound healing rate at 48 h for each treatment group. e) Number of junctions in HUVEC networks formed after treatment with each MN patch. f) Total segment length in HUVEC networks for each treatment. Data represented mean ± SEM from *n* = 3 independent experiments. *P* value: ns means no significance, ^*^ *p* < 0.05, ^**^ *p* < 0.01, ^***^ *p* < 0.001, and ^****^ *p* < 0.0001.

Human umbilical vein endothelial cells (HUVECs, Procell Life Science & Technology Co., Ltd., China) and mouse embryonic fibroblast cells (NIH‐3T3, Procell Life Science & Technology Co., Ltd., China) were utilized to assess the cell migration and angiogenesis‐promoting abilities of HA‐PP MN patches. In the cell migration assays for HUVECs (**Figure** **6**a) and NIH‐3T3 cells (Figure S7a, Supporting Information), the groups containing PDA@ZIF‐8@AB NPs demonstrated significantly enhanced wound healing rates compared to the Control and HA‐PZ MN groups. Specifically, at 24 h, the wound healing rate for the HA‐PZ MN group was 29 ± 2%, while the healing rates for the HA‐PZA MN, HA‐PZA MN + NIR, HA‐PP MN, and HA‐PP MN + NIR groups were ≈40% (Figure 6c). At 48 h, the wound healing rate for the HA‐PZ MN group was 50 ± 3%, while the groups containing PDA@ZIF‐8@AB NPs showed healing rates exceeding 70% (Figure 6d). NIH‐3T3 cells exhibited a similar trend (Figure S7b, c, Supporting Information). These results highlighted that the enhanced effect was attributed to the H_2_ generation capability of PDA@ZIF‐8@AB NPs, which has been shown to facilitate cell migration and promote wound healing processes.

In the tube formation assay with HUVECs (Figure 6b), the HA‐PZA MN and HA‐PP MN groups, particularly under NIR irradiation, demonstrated more robust and well‐defined tubular structures. Quantitative analysis showed that the HA‐PZ MN group had a number of junctions of 12.0 ± 1.6 and total segment length of 620 ± 7, while the groups containing PDA@ZIF‐8@AB NPs had a number of junctions greater than 30 (Figure 6e) and total segment length exceeding 1200 (Figure 6f). This effect was primarily attributed to the H_2_ generation driven by PDA@ZIF‐8@AB NPs, which stimulated angiogenic activity.

### 2.11 Prolonged and Potent Analgesic Effect of HA‐PP MN Patches with NIR Activation for Postoperative Pain

Before initiating the formal analgesic studies, we assessed the potential skin effects of the photothermal response from the HA‐PZA MN patches, which showed the highest temperature rise. The patches were applied to the dorsal (Figure S8a, Supporting Information) and plantar skin (Figure S8b, Supporting Information) of rats and subjected to six intermittent NIR irradiation cycles. Observations recorded during and 48 h post‐irradiation showed no visible signs of skin damage, confirming that the photothermal effect of the HA‐PZA MN patches did not cause adverse effects on the skin.

**Figure 7:**
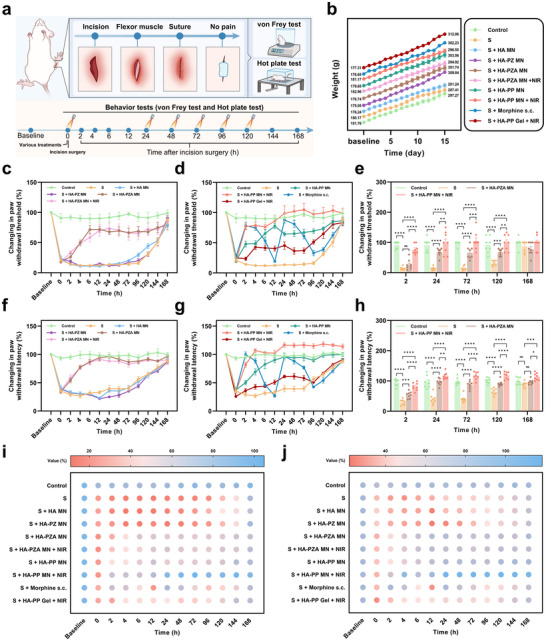
Behavioral tests for pain assessment following plantar incision surgery in rats and the effects of various MN treatments. a) Schematic of the plantar incision model and timeline of behavioral tests, including the von Frey filament and Hot plate assessments. b) Body weight changes of rats across 15 days post‐surgery in different treatment groups. Data were expressed as mean ± SEM (*n* = 16). c‐e) Percentage change in mechanical paw withdrawal threshold across treatment groups compared to baseline. f‐h) Percentage change in thermal withdrawal latency across treatment groups compared to baseline. i‐j) Bubble charts illustrating the time‐course improvement in mechanical and thermal pain thresholds across groups, with intensity changes visualized at each time point. All data were expressed as mean ± SEM (*n* = 8). *P* value: ns means no significance, ^*^ *p* < 0.05, ^**^ *p* < 0.01, ^***^ *p* < 0.001, and ^****^ *p* < 0.0001.

For the analgesic study, rats underwent plantar incision surgery to establish a postoperative pain model (**Figure** **7**a).^[^ ^33^ ^]^ Mechanical pain sensitivity was evaluated with von Frey filaments (Figure 7c–e; Figure S9a, b, Supporting Information), showing that the S and S + HA‐PZ MN group experienced an immediate reduction in paw withdrawal threshold to 21 ± 3% and 18 ± 3% of baseline, indicating heightened pain sensitivity. This sensitivity only began to subside from day 4 post‐surgery, returning to 88 ± 6% and 91 ± 4% of baseline by day 7. In contrast, the HA‐PZA MN, HA‐PZA MN + NIR, and HA‐PP MN groups, featuring the release of H_2_, demonstrated significant analgesic effects, with comparable pain relief lasting up to the fifth day post‐surgery, maintaining paw withdrawal thresholds of 68 ± 4%, 73 ± 11%, and 74 ± 3%, respectively. Notably, the HA‐PP MN + NIR group exhibited the most potent analgesic effect, reaching near‐complete pain threshold recovery within 24 h post‐surgery and maintaining a high paw withdrawal threshold of 100 ± 9% on day 5. This robust analgesic effect was attributed to the combination of H_2_ release from PDA@ZIF‐8@AB NPs and NIR‐triggered release of QX‐314, working synergistically to inhibit nociceptive signaling and improve pain outcomes. In comparison, the HA‐PP Gel + NIR group showed significantly weaker pain relief, indicating lower transdermal delivery efficiency and drug utilization compared to the MN formulation. For clinical comparison, the analgesic effect of a single subcutaneous morphine injection lasted only 4 h. Although the 3 mg kg^−1^ morphine dose significantly reduced pain sensitivity, the level of pain relief was not as effective as the HA‐PP MN + NIR group. With repeated injections, the analgesic effect of morphine slightly diminished.

Thermal pain sensitivity tests further supported these findings (Figure 7f–h; Figure S9c, d, Supporting Information). In the S group, the thermal pain threshold decreased immediately post‐surgery to 36 ± 3%, indicating abnormal thermal nociception. The HA‐PP MN + NIR group consistently showed increased paw withdrawal latency on the hot plate, starting at 2 h post‐surgery with a threshold of 82 ± 5%, and maintaining an elevated threshold above baseline up to day 7 (114 ± 4%). This sustained reduction in thermal sensitivity demonstrated effective pain relief compared to the S group. The movies captured the thermal pain testing of the S and HA‐PP MN + NIR groups 24 h post‐surgery, showcasing the marked differences in pain sensitivity (Movies S1–S6, Supporting Information). In groups with only PDA@ZIF‐8@AB NPs or one additional condition (PCL@QX‐314 MSs or NIR irradiation), analgesic effects in thermal pain also persisted for over five days, though with reduced intensity. This suggested that while PDA@ZIF‐8@AB's H_2_ generation contributed significantly to pain relief, the combination of NIR‐triggered QX‐314 release in the HA‐PP MN + NIR group maximized the analgesic effect. The bubble plots (Figure 7i,j) provided a visual representation of the analgesic effects of different treatments for mechanical pain (Figure 7i) and thermal pain (Figure 7j), highlighting the superior pain relief in the HA‐PP MN + NIR group.

Throughout the 15‐day study, the rats’ body weights were monitored to assess general health (Figure 7b). All groups showed steady weight gain, indicating that the treatments, including the NIR‐triggered HA‐PP MN patches, did not adversely affect the health or growth of rats. These findings collectively underscored the potential of HA‐PP MN patches with NIR, to deliver prolonged and controlled postoperative pain relief safely.

### 2.12 Suppression of Pain Pathway Activation by HA‐PP MN Patches with NIR

Studies confirm that 24 h post‐surgery is a critical phase for pain pathway activation and related gene expression.^[^ ^34^ ^]^ Our behavioral findings also showed significant analgesic effects of HA‐PP MN patches at this point. Thus, we selected this time to investigate the pain‐suppressive mechanisms induced by MN patches.

**Figure 8:**
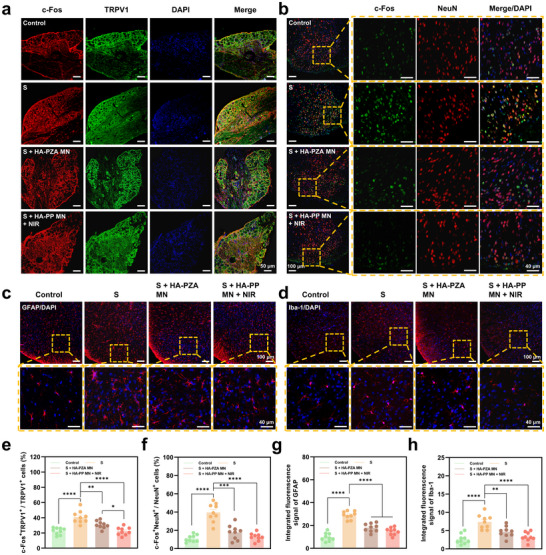
Immunofluorescence analysis of neuronal and glial activation in DRG and spinal cord following MN treatments. a) Representative immunofluorescence images showing c‐Fos and TRPV1 expression in DRG neurons under different treatment conditions. b) Representative immunofluorescence images for c‐Fos and NeuN in the SG area of spinal cord dorsal horn under different treatment conditions. c) GFAP immunostaining in the spinal cord dorsal horn revealed astrocyte activation across groups. d) Iba‐1 immunostaining in the spinal cord dorsal horn demonstrated microglial activation across groups. e‐h) Quantification of c‐Fos⁺TRPV1⁺/TRPV1⁺ (e), c‐Fos⁺NeuN⁺/NeuN⁺ (f) cell percentages, and integrated fluorescence intensities for GFAP (g) and Iba‐1 (**h)** across groups, respectively. All data were expressed as mean ± SEM (*n* = 9 slices from three rats). *P* value: ^*^ *p* < 0.05, ^**^ *p* < 0.01, ^***^ *p* < 0.001, and ^****^ *p* < 0.0001.

In the L4/L5 DRG, we analyzed c‐Fos and TRPV1 as markers associated with nociceptive neuron activation (**Figure** **8**a,e; Figure S10a, Supporting Information). TRPV1, a key receptor in pain transmission, is highly expressed in DRG nociceptive sensory neurons and plays an essential role in relaying peripheral pain signals to the central nervous system.^[^ ^35^ ^]^ C‐Fos serves as an indicator of neuronal activity,^[^ ^36^ ^]^ with increased c‐Fos^+^/TRPV1^+^ expression denoting heightened sensory neuron response to painful stimuli. Compared to the Control group, increased c‐Fos/TRPV1 co‐positive neurons in the S group indicated heightened nociceptive activation post‐incision (22.4 ± 1.5% vs 41 ± 3%). HA‐PZA MN and HA‐PP MN + NIR treatments significantly reduced this activation, with the HA‐PP MN + NIR group showing the most pronounced effect (22.2 ± 1.7%), suggesting effective inhibition of pain signal transmission.

In the substantia gelatinosa (SG) region of the spinal cord, we analyzed c‐Fos and NeuN as markers of central pain processing (Figure 8b,f; Figure S10b, Supporting Information). The SG region is pivotal for relaying and modulating nociceptive input from the periphery to higher brain centers.^[^ ^37^ ^]^ Elevated co‐expression of these markers in the S group (32 ± 4%) indicated central sensitization and enhanced transmission of pain signals. Both HA‐PZA MN and HA‐PP MN + NIR groups significantly lowered c‐Fos and NeuN expression in the SG, with HA‐PP MN + NIR showing the strongest inhibition (12.7 ± 1.4%), suggesting reduced central sensitization.

Additionally, we assessed glial cell activation by staining GFAP (astrocyte marker, Figure 8c,g) and Iba‐1 (microglial marker, Figure 8d,h) in the dorsal horn of the spinal cord, as astrocytes and microglia are involved in neuroinflammatory processes that exacerbate pain.^[^ ^38^ ^]^ Elevated GFAP and Iba‐1 expression in the S group reflected neuroinflammatory activation consistent with pain sensitization. Both HA‐PZA MN and HA‐PP MN + NIR treatments, particularly the HA‐PP MN + NIR group, significantly reduced GFAP and Iba‐1 levels. The decreased glial activation indicated that HA‐PP MN + NIR not only suppressed neuronal activation but also inhibited neuroinflammatory pathways involved in pain amplification.

Together, these findings suggested that HA‐PP MN patches, particularly with NIR activation, effectively modulated both peripheral and central nociceptive pathways, offering a promising strategy for targeted, sustained postoperative pain relief.

### 2.13 Accelerated Wound Healing Induced by the H_2_ Released from MN Patches

**Figure 9:**
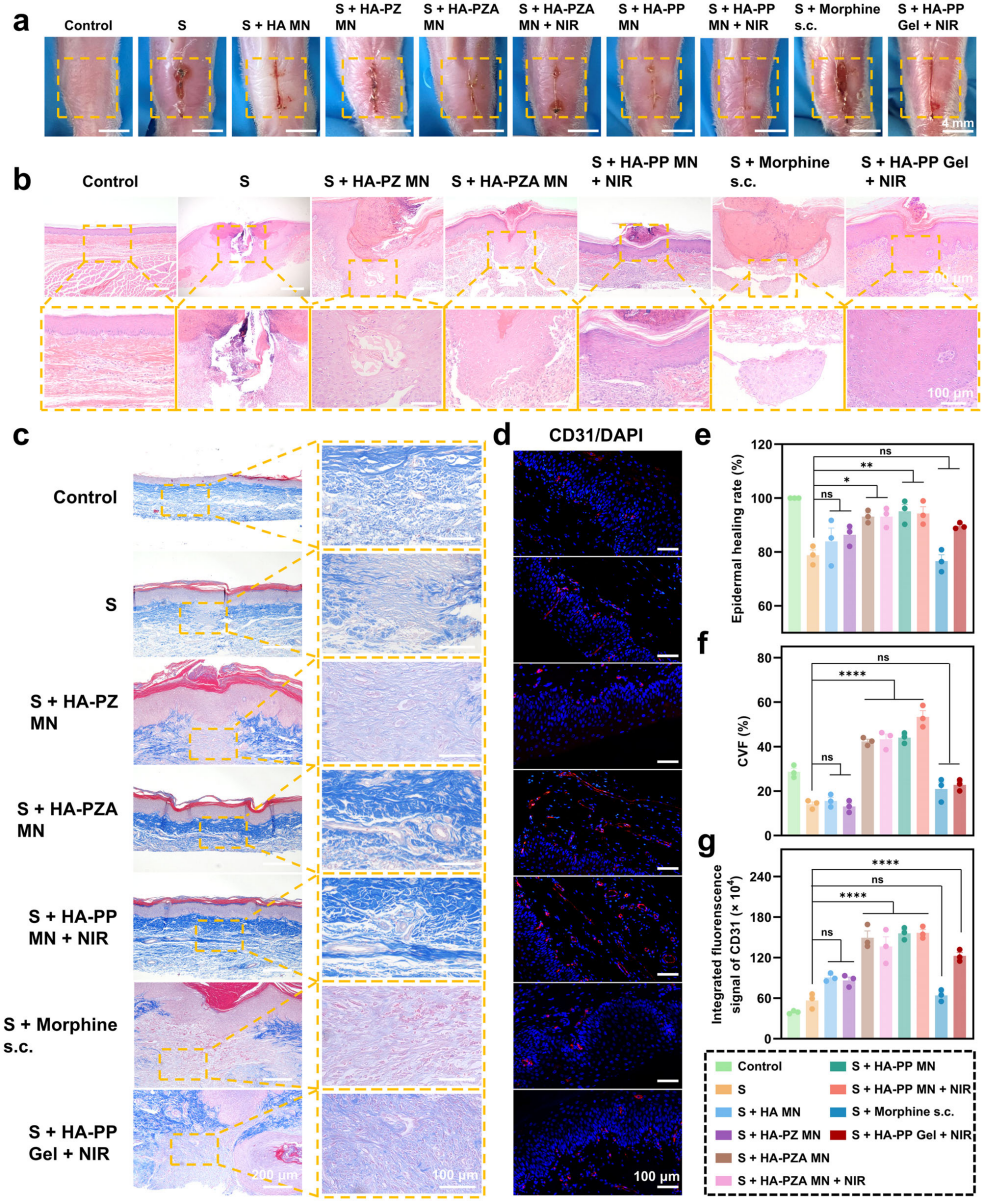
Evaluation of wound healing, collagen deposition, and angiogenesis following MN treatments. a) Representative images of wound healing across different treatment groups, showing wound closure progression. b) H&E‐stained tissue sections of the wound area. c) Masson's trichrome staining for collagen fibers in wound tissues. d) Immunofluorescence staining for CD31 in wound sections to assess angiogenesis. e‐g) Quantification of epidermal healing rate (e), CVF (f), and integrated fluorescence intensity of CD31 (g) across groups. All data were expressed as mean ± SEM (*n* = 3). *P* value: ns means no significance, ^*^ *p* < 0.05, ^**^ *p* < 0.01, and ^****^ *p* < 0.0001. CVF: collagen volume fraction.

Daily photographic observations of the surgical wounds demonstrated that all H_2_‐releasing MN‐treated groups, including HA‐PZA MN, HA‐PZA MN + NIR, HA‐PP MN, and HA‐PP MN + NIR, achieved more rapid wound closure compared to the S, HA MN, HA‐PZ MN, HA‐PP Gel + NIR, and Morphine s.c. groups, which showed slower healing (**Figure** **9**a).

On day 5, H&E staining highlighted that H_2_‐releasing MN patches facilitated more organized re‐epithelialization and tissue recovery at the incision site, with reformed epithelial layers and well‐structured tissue observed in these groups (Figure 9b,e). Notably, this effect was absent in the HA‐PZ MN group, underscoring the role of H_2_ generation in promoting effective wound repair.

By day 7, Masson's trichrome staining showed robust collagen deposition in the H_2_‐generating groups, indicating enhanced tissue regeneration (Figure 9c). Collagen organization was markedly improved, which supported better structural recovery in the wound area (Figure 9f). This trend suggested that H_2_ release aided in the collagen synthesis necessary for wound strength and stability. CD31 immunofluorescence staining further revealed an increase in microvascular density in the wounds treated with H_2_‐releasing MNs by day 7, pointing to a significant pro‐angiogenic effect that supported sustained tissue repair (Figure 9d,g). This enhanced vascularization was likely to promote nutrient delivery and waste removal, key factors in effective wound healing.

### 2.14 Transcriptome Analysis of DRG with H_2_‐Releasing MN Patches Applied After Surgery

**Figure 10:**
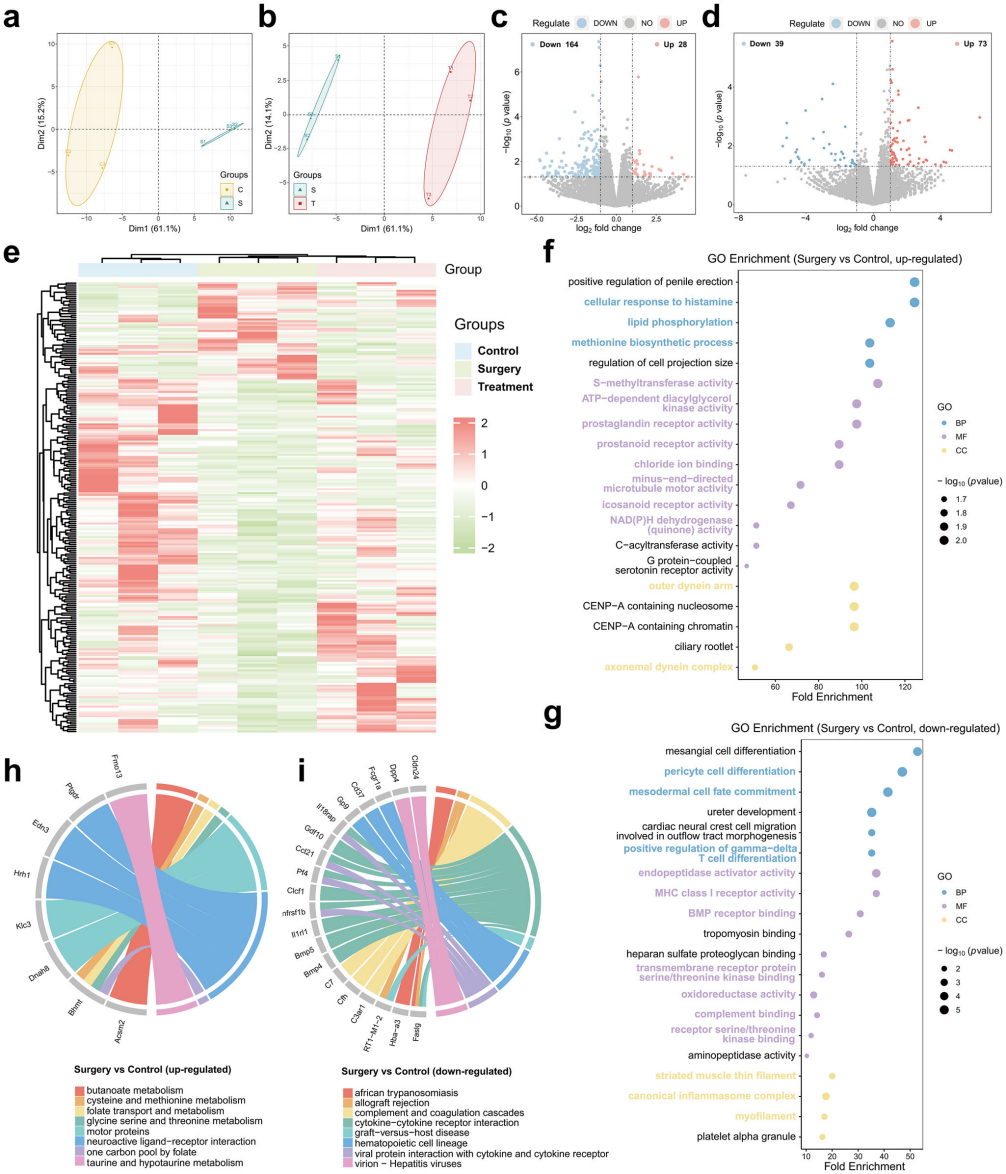
Transcriptomic analysis of DRG tissues from Control, Surgery, and Treatment groups. a‐b) PCA plots showing the distribution of samples among the Control, Surgery, and Treatment groups. c‐d) Volcano plots highlighting differentially expressed genes between the Surgery vs Control groups (c) and the Treatment vs Surgery groups (d). e) Heatmap of gene expression profiles. f) GO enrichment analysis for the Surgery vs Control comparison, highlighting up‐regulated pathways. g) GO enrichment analysis for the Surgery vs Control comparison, showing significant down‐regulated pathways. h) KEGG pathway enrichment analysis of differentially expressed genes in the Surgery vs Control comparisons (up‐regulated). i) KEGG pathway enrichment analysis of differentially expressed genes in the Surgery vs Control comparisons (down‐regulated). Data were presented from *n* = 3 biological replicates per group. Treatment group: group with H_2_‐releasing MN patches applied after surgery.

To elucidate the potential molecular mechanisms underlying the analgesic and wound‐healing effects of H_2_ released from MN patches, RNA‐seq was performed on L4/5 DRG tissues collected 24 h post‐surgery from three groups: Control (C group), Surgery (S group), and Treatment (T group, S + HA‐PZA MN). Considering the analgesic properties of QX‐314 are well‐established,^[^ ^20^ ^]^ this transcriptome study just focused on the specific role of H_2_ in postoperative pain relief and tissue repair. Sequencing analysis yielded consistently high mapping rates across all groups, with an average exceeding 97%, ensuring data robustness and reliable transcript alignment (Table S1, Supporting Information). Principal component analysis (PCA) demonstrated a clear separation between the Control, Surgery, and Treatment groups, indicative of distinct transcriptional shifts associated with surgical trauma and the reparative influence of H_2_‐releasing MN treatment (**Figure** **10**a,b).

Differential expression analysis revealed significant transcriptomic changes across conditions. In the comparison between Surgery and Control groups, 192 genes were significantly differentially expressed, with 164 down‐regulated and 28 up‐regulated, reflecting substantial transcriptomic alterations due to surgical stress (Figure 10c). When examining differences between the Treatment and Surgery groups, 112 genes exhibited significant regulation, with 73 up‐regulated and 39 down‐regulated, emphasizing the impact of H_2_ release on the DRG transcriptome in response to surgical insult (Figure 10d). A comprehensive heatmap of gene expression profiles further illustrated these differences, showing distinct clustering patterns that underscored the unique transcriptional landscapes associated with each experimental condition (Figure 10e).

**Figure 11:**
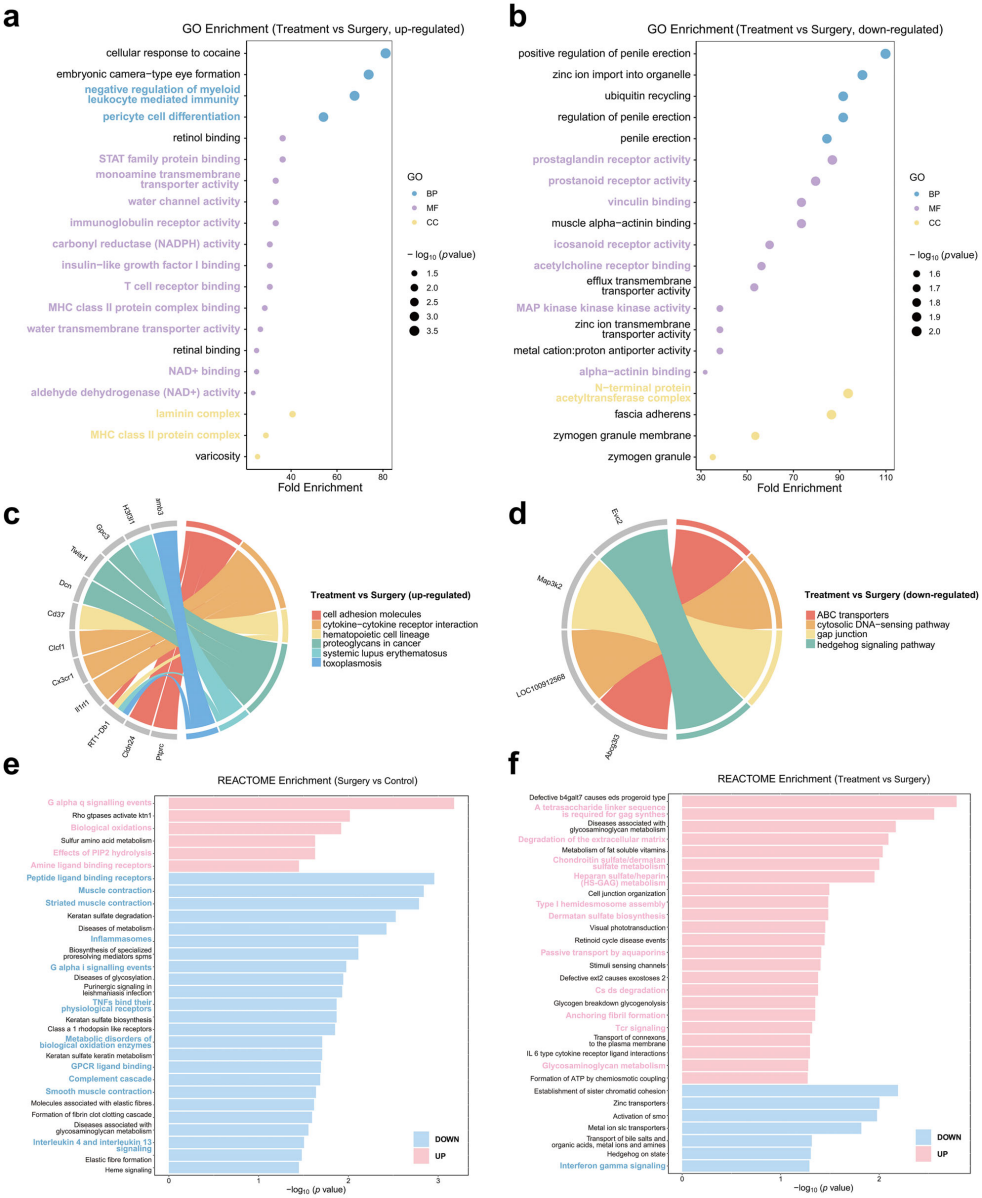
Enrichment analyses comparing Surgery vs Control groups and Treatment vs Surgery groups. a) GO enrichment analysis for the Treatment vs Surgery comparison, highlighting up‐regulated pathways. b) GO enrichment analysis for the Treatment vs Surgery comparison, showing significant down‐regulated pathways. c) KEGG pathway enrichment analysis of differentially expressed genes in the Treatment vs Surgery comparisons (up‐regulated). d) KEGG pathway enrichment analysis of differentially expressed genes in the Treatment vs Surgery comparisons (down‐regulated). e) Reactome enrichment analysis for the Surgery vs Control groups. f) Reactome enrichment analysis for the Treatment vs Surgery groups. Data were presented from *n* = 3 biological replicates per group.

Subsequently, we performed functional annotation of the differentially expressed genes (DEGs) across the groups. Gene Ontology (GO), Kyoto Encyclopedia of Genes and Genomes (KEGG), and Reactome pathway analyses were conducted based on the identified DEGs. The transcriptomic comparison between the Surgery and Control groups highlighted key early responses to surgical stress. The GO analysis revealed significant alterations in pathways associated with inflammation and pain, receptor and signal transduction, immune response and cell differentiation, metabolic regulation and enzyme activity, and muscle and cytoskeletal functions (Figure 10f,g). Specifically, pathways such as cellular response to histamine, prostaglandin receptor activity, icosanoid receptor activity and S‐methyltransferase activity were up‐regulated in the Surgery group, suggesting enhanced inflammatory and pain signaling (Figure 10f). In contrast, MHC class I receptor activity, receptor serine/threonine kinase binding, positive regulation of gamma‐delta T cell differentiation, and myofilament were down‐regulated (Figure 10g). KEGG pathway analysis identified the neuroactive ligand‐receptor interaction pathway as up‐regulated (Figure 10h), while the cytokine‐cytokine receptor interaction and hematopoietic cell lineage pathways were down‐regulated (Figure 10i). Reactome analysis further revealed the up‐regulation of signal transduction and receptor‐related pathways, such as G alpha q signaling events and effects of PIP2 hydrolysis. Additionally, pathways associated with oxidative stress, such as biological oxidations, were activated. Changes in inflammation regulation were observed, including alterations in inflammasome activity and TNF receptor binding, along with modifications in muscle function, as evidenced by the modulation of muscle contraction (**Figure** **11**e). In summary, surgical stress significantly impacted tissue inflammation, pain signaling, and cell function, thereby influencing the overall healing and recovery process.

To explore how H_2_‐releasing MNs control postoperative pain and accelerate wound healing, we analyzed transcriptomic differences between the Treatment and Surgery groups. This analysis specifically focused on the therapeutic effects of H_2_. The GO analysis revealed significant alterations in pathways associated with inflammation, pain, receptor binding and signal transduction, immune response, metabolic and redox processes, and cell differentiation. Notably, pathways involved in pericyte cell differentiation, monoamine transmembrane transporter activity, NADPH activity, NAD^+^ binding, water channel activity, and MHC class II protein complex were up‐regulated in the Treatment group (Figure 11a). In contrast, pathways such as prostaglandin receptor activity, icosanoid receptor activity, acetylcholine receptor binding, and MAPK kinase activity were down‐regulated, suggesting an inhibition in pain and inflammatory signaling (Figure 11b). KEGG pathway analysis identified the up‐regulation of hematopoietic cell lineage and cytokine‐cytokine receptor interaction pathways in the Treatment group, reflecting a modulation of immune and inflammatory responses (Figure 11c,d). Reactome analysis highlighted several pathways related to extracellular matrix metabolism and immune signaling. Pathways such as glycosaminoglycan metabolism, extracellular matrix degradation, and dermatan sulfate biosynthesis were up‐regulated, indicating an enhanced wound healing process. Additionally, the up‐regulation of T cell receptor (TCR) signaling and passive transport by aquaporins pathways was observed, consistent with the GO analysis findings (Figure 11f). The above enrichment results suggested that H_2_‐releasing MNs alleviated postoperative pain and promoted wound healing by inhibiting inflammation and oxidative stress, suppressing pain signal transduction and nociceptive receptor activity, and enhancing tissue regeneration.

**Figure 12:**
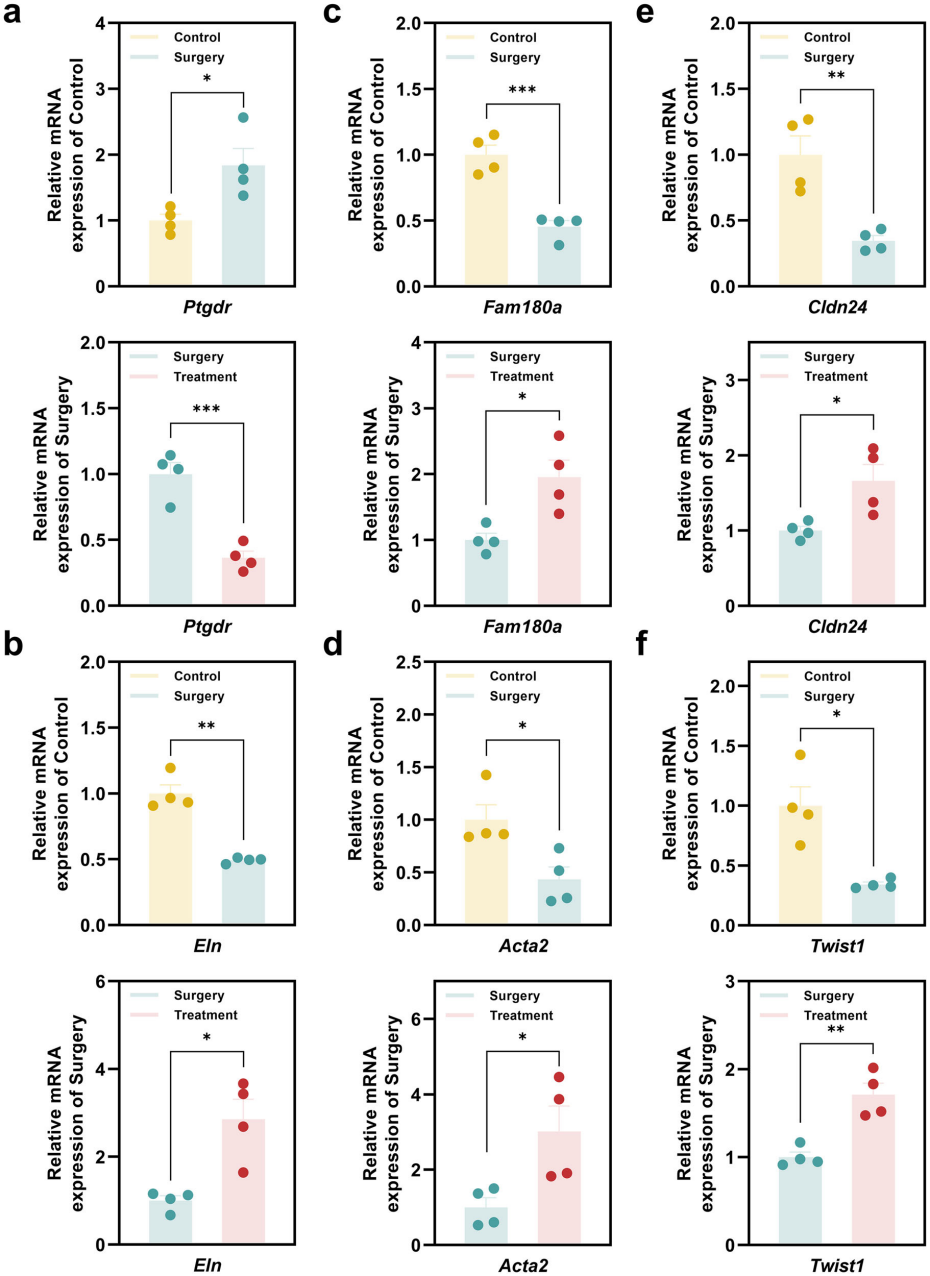
qPCR analysis of key genes associated with pain and wound healing. a‐f) Relative mRNA expression of selected genes in Control, Surgery, and Treatment groups. Genes analyzed included: Ptgdr (a), Eln (b), Fam180a (c), Acta2 (d), Cldn24 (e), and Twist1 (f). All data were presented as mean ± SEM (*n* = 4). *P* value: ^*^ *p* < 0.05, ^**^ *p* < 0.01 and ^***^ *p* < 0.001.

Based on the transcriptomic analysis, we further validated the sequencing results using RT‐qPCR for key differentially expressed genes. These genes were selected based on their relevance to our study, particularly in inflammation, pain signaling, and tissue healing processes. RT‐qPCR results confirmed the findings from RNA‐seq and showed that the regulatory trend of the selected genes was consistent with the sequencing results. For example, *Ptgdr*, which is involved in inflammatory pathways and is known to play a role in pain modulation, was significantly up‐regulated in the Surgery group compared to the Control group. Treatment with H_2_‐releasing MNs effectively reduced the *Ptgdr* expression, suggesting its potential role in alleviating postoperative pain (**Figure** **12**a). Similar trends were observed for genes related to tissue regeneration. The gene expression levels of *Eln*, *Fam180a*, and *Twist1* were significantly higher in the Treatment group, indicating the enhanced tissue repair and regeneration processes facilitated by H_2_ release (Figure 12b,c,f). Notably, the down‐regulation of *Acta2* and *Cldn24* in the Surgery group was reversed in the Treatment group, suggesting a restoration of cellular functions, particularly in cell‐cell adhesion and tissue integrity, suggesting an improvement in tissue homeostasis following H_2_ treatment (Figure 12d,e).

### 2.15 Evaluation of Biocompatibility and Biosafety of HA‐PP MN Patches

The biocompatibility and biosafety of MN patches were comprehensively assessed through in vitro and in vivo studies. In vitro cytotoxicity tests on HUVEC (Figure S12b‐d, Supporting Information) and NIH‐3T3 (Figure S13b‐d, Supporting Information) cells demonstrated excellent cell viability across varying concentrations of the patch components (HA, PDA@ZIF‐8@AB NPs, and PCL@QX‐314 MSs). The Calcein‐AM/PI staining indicated minimal cell death, with cell viability consistently exceeding 90% across all treatment groups, indicating that the materials did not adversely affect cellular integrity (Figures S12a and S13a, Supporting Information).

In vivo biocompatibility was evaluated using hemolysis assays and organ function markers. Hemolysis rates in all treated groups were well below the 5% safety threshold, suggesting minimal red blood cell lysis and excellent blood compatibility (Figure S14a, Supporting Information).^[^ ^39^ ^]^ Serum analyses for liver (ALT, AST) and kidney (BUN, CR) function revealed no significant differences between treatment and control groups, indicating that HA‐PP MN patches with NIR irradiation did not compromise major organ functions (Figure S14b‐e, Supporting Information). Furthermore, H&E staining of internal organs (heart, liver, spleen, lungs, and kidneys) showed no signs of inflammation, tissue damage, or morphological abnormalities, supporting the biocompatibility of the MN patches in vivo (Figure S15c, Supporting Information).

Upon application of MN patches to rat skin, the puncture sites were observed to completely close within 30 min, demonstrating the minimal invasiveness of the MNs (Figure S15a, Supporting Information). Subsequent H&E staining of the skin at application sites confirmed the absence of inflammatory infiltration or structural damage, further verifying the safety of repeated application and NIR‐triggered drug release (Figure S15b, Supporting Information). Collectively, these results underscored the excellent biosafety profile of HA‐PP MN patches, affirming their suitability for safe, transdermal therapeutic applications.

## 3 Discussion

In this study, we introduced a novel H_2_‐based therapeutic system delivered via intelligent photothermal MNs for postoperative pain management and wound healing. Using a dual‐phase MN system, we successfully achieved sustained hydrogen encompassment and controlled release of QX‐314 at the surgical sites. Our findings underscore the efficacy of H_2_ as a non‐traditional analgesic and its potential to accelerate wound healing. Besides, the photothermally triggered release of QX‐314 enables on‐demand pain relief, optimizing pain management based on individual patient pain thresholds.

Several studies have explored the use of H_2_ as a therapeutic agent, particularly for its antioxidant^[^ ^40^ ^]^ and anti‐inflammatory^[^ ^41^ ^]^ properties, which play a crucial role in pain management and tissue repair. Li et al.^[^ ^12^ ^]^ demonstrated that H_2_ reduced neuroinflammation and microglial activation in a mouse model by inhibiting the ASK1/JNK/p38/MMP9 signaling pathway, thereby alleviated postoperative pain. Similarly, Chen et al.^[^ ^42^ ^]^ utilized H_2_ as an antioxidant to alleviate chronic wound inflammation and promote healing. Our HA‐PP MN exhibits similar therapeutic effects and can provide effective analgesia for up to 5 days.

Our transcriptomic analysis provided deeper insights into the molecular mechanisms underlying the observed analgesic and regenerative effects of H_2_ therapies. The data revealed distinct biological pathways involved in the response to surgical injury and subsequent treatment. Surgical trauma led to inflammatory responses and oxidative stress, simultaneously upregulating pain‐related signaling pathways and receptor activities, such as prostaglandin receptor activity. This suggest that the inflammation and other responses triggered by surgical stimuli play a critical role in postoperative pain. In contrast, H_2_‐releasing MNs led to a shift in gene expression reflecting both analgesic and regenerative effects. The up‐regulation of antioxidant stress pathways in the Treatment group suggested that H_2_ may mitigate oxidative damage, promote tissue repair, and accelerate wound healing. Additionally, the up‐regulated pathways related to pericyte differentiation, NADPH activity, and extracellular matrix regulation suggested enhanced tissue repair and resolution of inflammation. Modulation of immune‐related pathways further highlighted the role of H_2_ in regulating inflammation. The down‐regulation of pain‐related signaling pathways, such as prostaglandin receptor activity and MAPK signaling, reinforced the analgesic potential of H_2_‐releasing MNs. Building on previous research, these findings emphasize the therapeutic potential of H_2_ in analgesia and tissue regeneration by inhibiting inflammation and oxidative stress.

Delivering H_2_ in a controlled and sustained manner, especially for localized applications^[^ ^43^ ^]^ are still challenging. In our study, we addressed this issue by using ZIF‐8 NPs as a scaffold for AB, allowing for controlled and sustained H_2_ release within the MN system. ZIF‐8 is a structurally stable and biocompatible metal‐organic framework (MOF) widely used in drug delivery systems.^[^ ^44^ ^]^ Previous research by Fang et al.^[^ ^45^ ^]^ showed that modifying ZIF‐8 could improve the stability and efficacy of anticancer drugs, further supporting its potential in biomedical applications. Notably, ZIF‐8's pH‐responsive degradation is particularly advantageous in postoperative acidic environments, where it facilitates the release of encapsulated drugs.^[^ ^46^ ^]^ In our system, the encapsulation of AB within ZIF‐8 NPs enabled sustained and localized H_2_ release around the surgical sites, overcoming the typical rapid diffusion associated with H_2_ therapies.

On‐demand analgesia strategies have become a focal point in recent pain management research. For example, Song et al.^[^ ^47^ ^]^ introduced a nanoparticle platform that provides sustained, on‐demand pain relief using moderate‐intensity ultrasound, while Reeder et al.^[^ ^48^ ^]^ developed a soft, biodegradable cooling device that achieves on‐demand analgesia by lowering nerve temperature. These systems allow for real‐time modulation of pain relief, pushing the boundaries of personalized pain management. QX‐314 represents a promising approach for on‐demand analgesia due to its permanent positive charge, which prevents it from crossing cell membranes.^[^ ^49^ ^]^ As a result, it must be delivered intracellularly, such as through activated ion channels, to exert its effects.^[^ ^50^ ^]^ Based on this property, we incorporated QX‐314 into the MN system, utilizing the photothermal responsiveness of PDA and the temperature‐sensitive properties of PCL MSs. This system enabled controlled, on‐demand release of QX‐314 under NIR irradiation, facilitating the entry of QX‐314 into neurons and providing localized pain relief at the surgical site. This approach, offering precise modulation of QX‐314 release in response to individual pain thresholds, represents a significant step forward in personalized pain management, offering a more targeted alternative to traditional methods.

MN transdermal delivery systems offer several advantages, including minimally invasive administration, high patient compliance, and controlled release capabilities.^[^ ^51^ ^]^ Recent studies have explored dual‐release systems to address diverse therapeutic needs. For instance, Gu et al.^[^ ^52^ ^]^ developed a glucagon patch with both immediate and biologically responsive release modes for the emergency treatment of severe hyperglycemia. Younas et al.^[^ ^53^ ^]^ created a MN‐based dual‐release system for rapid analgesic delivery combined with sustained antimicrobial release to promote wound healing. In our study, we developed a dual‐phase MN system that integrates both sustained and on‐demand release strategies. H_2_ was continuously released to reduce inflammation, relieve pain and promote tissue repair, while QX‐314 was released on‐demand for immediate, localized pain relief. This dual‐phase approach not only offers personalized pain management but also enhances the precision of postoperative care, marking a notable advancement over traditional analgesic strategies and facilitating more tailored therapeutic interventions.

While our system demonstrates promising results, there are still some limitations. First, the need for an external NIR device to trigger QX‐314 release limits the system's practicality and integration. An autonomous, fully integrated system would be more desirable for clinical use, and further development is needed to eliminate the reliance on external equipment. Additionally, while H_2_ delivery via ZIF‐8 NPs effectively reduces inflammation and promotes tissue regeneration, controlling the release rate over extended periods remains a challenge. The long‐term stability of H_2_ delivery, as well as the potential for local tissue saturation, require further optimization and investigation.

## 4 Conclusions

In conclusion, the smart photothermal‐responsive MN system developed in this study successfully integrated sustained H_2_ release and photothermal‐triggered QX‐314 delivery, achieving both long‐lasting analgesia and accelerated tissue repair. The ZIF‐8‐based hydrogen release system provided localized, long‐lasting anti‐inflammatory effects, while controlled QX‐314 release allowed for customized pain relief, addressing the limitations of conventional pain management strategies. These findings underscore the therapeutic potential of this dual‐action MN system, offering a personalized approach that meets the analgesic and regenerative needs in postoperative care. This technology not only holds promise for enhancing recovery protocols but also lays the groundwork for the future development of responsive drug delivery systems.

## 5 Experimental Section

### Materials

All reagents used in this study were of analytical grade and purchased from commercial suppliers. These included ammonia borane (AB, 97%, Aladdin, China), 2‐Methylimidazole (2‐MI, ≥98%, Aladdin, China), zinc nitrate hexahydrate (99%, Chronchem, China), dopamine hydrochloride (98%, Macklin, China), QX‐314 (≥98%, Sigma Aldrich, USA), hyaluronic acid (HA, 40–100 kDa, Macklin, China), polycaprolactone (PCL, Mn 80000, Sigma Aldrich, USA), polyvinyl alcohol (PVA, Mw 9000–10000, Sigma Aldrich, USA), sodium chloride (99.5%, Macklin, China), HA (10 kDa, Rhawn, China), methylene blue (≥98%, Macklin, China), chloroplatinic acid hexahydrate (Pt ≥ 37.5%, Aladdin, China), fluorescein sodium salt (70%, Aladdin, China), and rhodamine B (95%, Solarbio, China). DMEM and FBS were obtained from Procell (China). The Annexin V‐FITC/PI Apoptosis Kit was purchased from Elabscience (China), while both Cell Counting Kit‐8 (CCK‐8) and Calcein‐AM/PI Cell Viability/Cytotoxicity Assay Kit were sourced from Beyotime (China). The antibodies used in this study included TRPV1 (Affinity Biosciences, DF10320), NeuN (Abcam, ab177487), c‐Fos (Servicebio, GB12069), Iba‐1 (Abcam, ab283319), GFAP (Cell Signaling Technology, 3670S), Goat Anti‐Rabbit IgG H&L (FITC conjugated, Servicebio, GB22303), Goat Anti‐Mouse IgG H&L (Cy3 conjugated, Servicebio, GB21301), Goat Anti‐Rabbit IgG H&L (Cy3 conjugated, Servicebio, GB21303), and Goat Anti‐Mouse IgG H&L (FITC conjugated, Servicebio, GB22301).

### Synthesis of PDA@ZIF‐8@AB NPs

ZIF‐8@AB NPs were synthesized using a one‐step method.^[^ ^54^ ^]^ Zinc nitrate hexahydrate (104.125 mg) was dissolved in 700 µL of deionized water and sonicated for 5 min at room temperature to ensure complete dissolution. Meanwhile, 2‐MI (2.05 g) and AB (50 mg) were dissolved in 10 mL of deionized water, and the mixture was stirred at 500 rpm for 5 min to create a homogeneous solution. The zinc nitrate solution was then added dropwise to the 2‐MI‐AB mixture while stirring at 1500 rpm at room temperature for 30 min. After this, the resulting mixture was centrifuged at 10 000 rpm for 15 min to collect the precipitate, which was washed with deionized water, yielding ZIF‐8@AB. The precipitate was then resuspended in 10 mL of deionized water, and the pH was adjusted to 8.5 using Tris‐HCl buffer. Subsequently, 10 mg of dopamine hydrochloride was added, and the solution was stirred at 1500 rpm for 2 h at room temperature. After 2 h, the resulting product was collected by centrifugation at 10 000 rpm for 15 min, washed three times with deionized water, and then freeze‐dried under vacuum for 48 h to obtain PDA@ZIF‐8@AB NPs. The blank NPs were prepared with the same procedures without adding AB to the 2‐MI solution.

### Preparation of PCL@QX‐314 MSs

Drug‐loaded MSs were prepared using an emulsification‐solvent evaporation method.^[^ ^55^ ^]^ In brief, 30 mg of QX‐314 and 20 mg of HA (40‐100 kDa) were dissolved in 1 mL of deionized water to form the inner aqueous phase (W1). Separately, 250 mg of PCL was dissolved in 5 mL of dichloromethane to create the organic phase (O). The two solutions were then emulsified for the first time using an ultrasonic processor under ice bath conditions, with a power setting of 150 W for 1 min, resulting in a water‐in‐oil (W/O) primary emulsion. Next, the primary emulsion was quickly transferred to 10 mL of a PVA (2%, w/v) and sodium chloride (1%, w/v) solution (W2). The mixture was hand‐shaken at a speed of 100 shakes min^−1^ for 1 min, resulting in a water‐in‐oil‐in‐water (W1/O/W2) multiple emulsion. The multiple emulsion was then rapidly poured into 20 mL of a PVA (0.2%, w/v) solution and stirred at 300 rpm at room temperature for 4 h to allow for the evaporation of dichloromethane, leading to the solidification of the polymer matrix and the formation of MSs. The products were collected by centrifugation at 6000 rpm for 15 min, washed three times with deionized water, and then freeze‐dried under vacuum for 48 h to obtain PCL@QX‐314 MSs. The preparation steps for the blank MSs were identical, except that no QX‐314 was added to the inner aqueous phase (W1).

### Characterization of PDA@ZIF‐8@AB NPs

The microstructure of the nanoparticles was characterized using TEM (JEOL, JEM‐2100). The particle size, polydispersity index (PDI), and zeta potential of the samples were measured using a Malvern Zetasizer (Malvern Instruments, Zetasizer Nano ZSP), with samples prepared in deionized water at a concentration of 1 mg mL^−1^. The crystal structure of the nanoparticles was analyzed by XRD (PANalytical, XPert Pro) over a 2 ϴ range from 10° to 80°. FT‐IR was conducted using an FT‐IR spectrometer (Thermo Fisher Scientific, Nicolet iS50 FTIR). Samples were prepared using the KBr pellet method to examine interactions between functional groups in the samples. UV‐vis spectra of the prepared samples were recorded using a UV‐Vis spectrophotometer (Shimadzu, UV‐2600). XPS (Thermo Fisher Scientific, ESCALAB Xi+) was utilized to identify the types and chemical states of elements present on the materials’ surfaces. XPS measurements were conducted both before and after the etching process. Prior to etching, surface elements were analyzed to obtain the composition of the original material. The etching depth was set to 20 µm, which was determined based on the particle sizes of ZIF‐8@AB NPs and PDA@ZIF‐8@AB NPs. After the etching process, XPS analysis was repeated to examine the internal elements of the NPs. Elemental mapping was performed using a field emission transmission electron microscope (JEOL, JEM‐F200). Finally, TGA (Mettler‐Toledo, TGA2 / DSC3) was conducted to determine the drug loading capacity.

### Measurement of Stability and Biocompatibility of PDA@ZIF‐8@AB NPs

ZIF‐8@AB and PDA@ZIF‐8@AB NPs were separately suspended in four different solvents: deionized water, PBS, DMEM, and FBS. The samples were allowed to sit at 4 °C, and their dispersibility was observed through photographs taken at 12 and 24 h. Additionally, DLS was used to measure the particle size, allowing for the analysis of the impact of PDA modification on material stability.

To evaluate the biocompatibility of the nanoparticles, NIH‐3T3 cells were utilized. The cells were cultured in DMEM supplemented with 10% FBS, penicillin (100 units mL^−1^), and streptomycin (100 µg mL^−1^). Different concentrations of ZIF‐8@AB and PDA@ZIF‐8@AB NPs were co‐cultured with the cells for 24 h. Subsequently, the treated cells were stained using an Annexin V‐FITC/PI apoptosis kit according to the manufacturer's instructions.

### Acid‐Responsive Degradation and H_2_ Release Behavior of PDA@ZIF‐8@AB NPs In Vitro

To investigate the degradation characteristics of the nanoparticles in acidic environments, PDA@ZIF‐8@AB NPs were suspended in PBS at pH values of 7.4 and 6.8, maintaining equal concentrations. The entire system was placed on a shaker (150 rpm) at 37 °C. At specific time points, namely days 1, 2, 5, and 7, 100 µL samples were collected from the PBS. After drying at room temperature, these samples were analyzed using TEM to evaluate the extent of degradation.

H_2_ has the capability to rapidly reduce MB to colorless reductive MB under the catalysis of Pt nanocrystals.^[^ ^56^ ^]^ Therefore, MB‐Pt probe was used to indirectly detect H_2_ release. A probe solution was prepared containing MB at a concentration of 300 µg mL^−1^ and Pt nanocrystals (3 nm) at a concentration of 13.9 µg mL^−1^. This concentration ensured that the MB remained within the detection range of the UV‐vis spectrophotometer and could fully absorb all H_2_ released from PDA@ZIF‐8@AB NPs. The absorbance of MB was measured at 664 nm. 10 mg of PDA@ZIF‐8@AB NPs were dispersed in 4 mL of the MB‐Pt probe solution at different pH values (7.4 and 6.8) and incubated on a shaker (150 rpm) at 37 °C. The release of hydrogen was quantified by monitoring the absorbance changes of the solution at predetermined time points (0, 1, 2, 4, 6, 12, 24, 48, 72, 96, 120, 144, and 168 hours), while the color changes of the solution were photographed. Subsequently, an in vitro cumulative release curve was plotted based on the calculated data.

### Evaluation of the Photothermal Properties of PDA@ZIF‐8@AB NPs

To evaluate the photothermal properties of PDA@ZIF‐8@AB NPs, a series of experiments were performed. First, PBS solutions containing different concentrations of PDA@ZIF‐8@AB NPs (0, 250, 500, and 800 µg mL^−1^) were irradiated with an 808 nm NIR laser at a power density of 1.0 W cm^−2^ for 8 min. Temperature changes were continuously monitored using a thermal infrared imager (FLIR SYSTEMS AB, FLIRSC7700M), capturing infrared images at 1‐minute intervals for each sample. Furthermore, the 500 µg mL^−1^ solution of PDA@ZIF‐8@AB was subjected to various laser power densities (0.5, 1.0, and 1.5 W cm^−2^) for 8 min, with temperature variations similarly monitored. To assess the stability of the photothermal response, the PDA@ZIF‐8@AB suspension underwent cyclic NIR irradiation, focusing on the temperature fluctuations during exposure. The 500 µg mL^−1^ solution was irradiated with the 808 nm laser at a power density of 1.5 W cm^−2^ for 8 min, followed by cooling to room temperature with the laser turned off. This process was repeated five times to analyze photothermal stability. The photothermal conversion efficiency (η) was calculated using established methods.^[^ ^57^ ^]^

### Characterization of PCL@QX‐314 MSs

The morphology of the MSs was analyzed using SEM, and particle sizes were measured using the ImageJ software based on the SEM images. Ion‐slicing technology (TESCAN, TESCAN Amber equipped with C‐ToF‐SIMS) was employed to obtain cross‐sections of the PCL@QX‐314 MSs for observing the internal structure. Structural characterization of the MSs was conducted using XRD and FT‐IR, with the detection methods being the same as those used for the NPs. The thermal properties of the samples were evaluated using TGA and DSC (Mettler‐Toledo, TGA2/DSC3), with TGA employed to calculate the drug loading capacity and DSC used to evaluate the thermal transitions of the materials.

### Thermal Response Characteristics and QX‐314 Release Behavior of PCL@QX‐314 MSs In Vitro

Equal concentrations (500 µg mL^−1^) of PCL@QX‐314 MSs and PDA@ZIF‐8@AB NPs were dispersed in PBS solution and incubated on a shaker at 150 rpm and 37 °C. For the NIR irradiation group, samples were exposed to NIR irradiation (1.0 W cm^−2^ for 5 min) at designated time points (24, 48, 72, 96, 120, and 144 h), while the control group remained unirradiated. Samples from the NIR irradiation group were collected after 0, 2, 4, and 6 cycles of laser exposure, while samples from the control group were collected at 0, 3, 5, and 7 days. The morphological changes of the MSs were examined using SEM.

To investigate the release behavior of QX‐314 at varying pH levels (7.4 and 6.8), 10 mg of PCL@QX‐314 MSs and an equal amount of PDA@ZIF‐8@AB NPs were added to each PBS solution. The mixtures were then incubated on a shaker at 150 rpm and 37 °C. For the NIR irradiation group, samples were exposed to NIR irradiation (1.0 W cm^−2^ for 5 min) at specific time points (24, 48, 72, 96, 120, and 144 h). At predetermined intervals (0, 1, 2, 4, 6, 12, 24, 48, 72, 96, 120, 144, and 168 h), 200 µL of supernatant was collected from each system and stored at 4 °C, with 200 µL of fresh PBS added to maintain volume. The samples were analyzed using a UV‐vis spectrophotometer at 193 nm to calculate the cumulative release rate of QX‐314.

### Fabrication and Characterization of MN Patches

PDA@ZIF‐8@AB NPs and PCL@QX‐314 MSs (3%, w/v) were dispersed in a Tris‐HCl buffer solution (pH 8.0) containing 18% (w/v) HA to obtain the matrix solution. This solution was injected into polydimethylsiloxane (PDMS, Dow Corning, USA) molds and vacuumed for 2 h, then the molds were transferred to a high‐speed centrifuge and centrifuged at 4200 rpm for 5 min. After removing the excess polymer solution, additional blank HA matrix solution was injected into the molds, which were then subjected to vacuum treatment for 4 h and dried in a 40 °C oven for 48 h. The resulting dual‐phase MN patches (HA‐PP MN) were subsequently peeled off from the PDMS molds. HA‐PZ MN loaded only PDA@ZIF‐8 NPs, while HA‐PZA MN contained PDA@ZIF‐8@AB NPs without any MSs, and HA MN was prepared from a blank matrix solution. Rhodamine B was used as a model drug for the NPs, and fluorescein sodium served as a model drug for the MSs, allowing for fluorescence visualization of the MN patches. Furthermore, we also prepared a gel formulation (HA‐PP Gel) using the same concentration of HA (18%, w/v), which loaded equal amounts of PDA@ZIF‐8@AB NPs and PCL@QX‐314 MSs. This was done to compare the transdermal efficiency and therapeutic effects between the MNs and the conventional formulation.

The morphology of the MN patches was observed using an optical microscope (LEICA, TL3000 Ergo) and SEM. Mechanical strength was assessed with a force measurement system (Mark‐10, ESM303). A single MN was fixed vertically on a steel platform, and pressure was applied at a constant speed of 2.1 mm min^−1^. The pressure‐displacement curve was recorded to evaluate the mechanical response of the MN. To evaluate the skin penetration capability of the HA‐PP MNs, isolated porcine skin was used in experiments and observed under an optical microscope after applying the MNs. Subsequently, the porcine skin was embedded and sliced into 12 µm thick sections using a cryostat microtome (Thermo Fisher Scientific, HM525 NX) for analysis under an inverted fluorescence microscope (Olympus, IX73).

### Photothermal Properties and Laser‐Controlled Drug Release of MN Patches

To assess the NIR irradiation activation of PDA@ZIF‐8@AB NPs in the MN patches, the HA MN patches, HA‐PZA MN patches, and HA‐PP MN patches were exposed to an 808 nm NIR laser (1.0 W cm^−2^) for 5 min. The temperature changes and thermal images of the MN patches were collected and monitored by a thermal infrared imager in real time.

To examine the potential influence of PDA's photothermal effect on drug release from NPs and MSs, fluorescent HA‐PP MN patches were inserted into a skin‐mimicking agarose gel (1.4%, w/v). The patches underwent laser irradiation for 5 min, followed by a 60‐minute cooling period with the laser off. This “on/off” light cycle was repeated for a total of 6 times. The diffusion of the two model drugs within the agarose gel was then evaluated using an inverted fluorescence microscope.

To evaluate the retention and sustained release of NPs and MSs in the local tissue microenvironment, fluorescent HA‐PP MN patches were applied for in vivo puncture testing. To minimize interference from factors like wound exudates on visualization, non‐surgical rat paw skin was used. During MN preparation, the matrix pH was adjusted to 6.8, creating a local acidic environment upon in vivo dissolution, which triggers the degradation and release of NPs. For the NIR irradiation group, the samples were exposed daily to an 808 nm NIR laser (1.0 W cm^−2^) for 5 min. Fluorescence images were captured over a 5‐day period, and fluorescence analysis was used to assess the duration of NPs and MSs retention and release in the local tissue.

### Promotion of Migration and Angiogenesis by MN Patches In Vitro

HUVEC and NIH‐3T3 cells were utilized to evaluate the impact of MN patches on cell migration. The culture was maintained in a humidified incubator at 37 °C with 5% CO_2_. Cells were seeded in 6‐well plates and cultured for 24 h. A 200 µL pipette tip was then used to create a uniform scratch in the center of each well. After washing with PBS to remove any floating cells, the cells were subjected to different conditions, divided into six groups: Control group, HA‐PZ MN group, HA‐PZA MN group, HA‐PZA MN with NIR laser group, HA‐PP group, and HA‐PP with NIR laser group. The MN patches were initially dissolved in DMEM containing 10% FBS. For the groups requiring NIR irradiation, the patches were subjected to 5 min of NIR exposure before being added to the wells. Cell migration was recorded at 0, 24, and 48 h.

To evaluate HUVEC tube formation, 100 µL of thawed Matrigel (Abwbio, 0827065) was added to each well of a 24‐well plate and incubated at 37 °C for 1 h to solidify. Then, 1.5 × 10^5^ HUVECs were seeded onto the Matrigel and exposed to various treatments. Tube formation was observed using an inverted fluorescence microscope, and live cells were visualized with a Calcein‐AM kit. ImageJ software was utilized for quantitative analysis of the tube structures.

### Development of Plantar Incision Model

All procedures were approved by the Experimental Animal Welfare Ethics Committee, Zhongnan Hospital of Wuhan University (Approval No: ZN2024100). Healthy male Sprague‐Dawley rats aged 6–8 weeks, sourced from China Three Gorges University, were kept in a controlled environment with humidity maintained at 40–60% and a temperature of 22 ± 2 °C. The animals were provided free access to food and water, kept on a 12‐hour light cycle, and their body weights were recorded daily during the experiment. All rats were randomly assigned to 10 groups (*n* = 8 per group). Except for the Control group, all underwent plantar incision surgery and received various treatments (S, S + HA MN, S + HA‐PZ MN, S + HA‐PZA MN, S + HA‐PZA MN + NIR, S + HA‐PP MN, S + HA‐PP MN + NIR, S + Morphine s.c., and S + HA‐PP Gel + NIR groups).

The plantar incision model was established following the previously described method.^[^ ^8^ ^]^ The rats were anesthetized via intraperitoneal injection of 2% sodium pentobarbital. After disinfection, a longitudinal incision of 1 cm was made on the left hind paw, positioned 2 mm from the heel, penetrating to the muscle while preserving the integrity of the muscle attachments at both ends. The skin was closed with 5‐0 sutures. The rats in the Control group received anesthesia, disinfection, and antibiotic treatment without undergoing any surgical procedures. After the recovery of the righting reflex, the appropriate treatment measures were immediately administered to each group. For the MN and gel groups, the treatments were applied directly to the incision site as a single treatment immediately post‐surgery. The morphine group received subcutaneous injections (3 mg kg^−1^) once a day around the incision site immediately after the surgery and within 3 days after the surgery. The NIR irradiation group was exposed to a 5‐minute daily laser treatment at the wound site for 6 days post‐surgery.

### Behavior Test for Postoperative Pain

Behavioral assessments for postoperative pain were conducted at specific intervals (baseline, 0, 2, 4, 6, 12, 24, 48, 72, 96, 120, 144, and 168 h after surgery). Mechanical pain sensitivity in the rats was evaluated using von Frey filaments. Prior to each session, the animals were allowed a 30‐minute acclimation period in the testing environment. A series of calibrated filaments with varying forces (1.0, 1.4, 2.0, 4.0, 6.0, 8.0, 10.0, and 15.0 g) were applied sequentially to the area around the plantar incision. Each filament was inserted into the tissue three times, with a 5‐minute interval between applications. The pressure was gradually increased until a withdrawal response was observed, with the force at which this occurred recorded as the paw withdrawal threshold. Additionally, thermal pain sensitivity was assessed. For three days prior to testing, rats were acclimatized to the hot plate for 30 min each day. During the test, the rats were placed on a heated surface set to 52.5 ± 0.5 °C, and the latency to withdraw or lick their paw was measured. Each rat underwent three trials, spaced 10 min apart, with a cut‐off time of 30 s implemented to prevent potential tissue damage.

### Immunofluorescence Staining

The L4/L5 DRG and spinal cord segments from the ipsilateral side (model side) of rats were collected 24 h post‐surgery for immunofluorescence staining. Samples were prepared as frozen sections, with the spinal cord cut to a thickness of 20 µm and the DRG to 10 µm. The expression levels of c‐Fos, TRPV1, NeuN, GFAP, and Iba‐1 were evaluated to investigate the activation of pain pathways. Positive neurons were quantified, and integrated fluorescence signal was calculated using ImageJ software.

### Assessment of Surgical Wound Healing and Histological Analysis

For a duration of seven days post‐surgery, photographs were taken daily to document the healing process of the surgical incision. On the fifth postoperative day, skin samples surrounding the plantar incision were collected for H&E staining to evaluate the healing of the damaged skin. Similarly, on the seventh postoperative day, samples were obtained for Masson's trichrome staining and CD31 immunofluorescence staining, aimed at assessing collagen deposition and microvascular density, respectively.

### RNA Sequencing and Data Analysis

RNA‐seq technology was applied to investigate the mechanisms by which H_2_ released from MN patches contributed to postoperative analgesia and wound healing. At 24 hours post‐surgery, the L4/5 DRG were harvested from the Control group (C group), Surgery group (S group), and the S+HA‐PZA MN group (Treatment, T group) for RNA‐seq library preparation and analysis. Each group contained three biological replicates. A total of 0.5 µg of RNA from each sample was used for library preparation, which was performed using the VAHTS mRNA Capture Beads 2.0 kit (Vazyme, China). Poly‐T oligo‐attached magnetic beads were used to purify mRNA. RNA integrity was assessed using the Agilent Bioanalyzer 2100 system (Agilent Technologies, USA). Library construction and quality control were carried out following standard protocols. Sequencing was performed on the Illumina NovaSeq 6000 platform (Illumina Inc., USA), with a paired‐end 150 bp (PE150) read length.

Raw data were processed using in‐house Perl scripts for quality control, including the removal of adaptor‐contaminated reads, low‐quality reads, and reads containing a high proportion of N bases. Clean reads were aligned to the mRatBN7.2 reference genome using HISAT2 v2.1.0,^[^ ^58^ ^]^ with genome and annotation files downloaded from the ENSEMBL database (http://ftp.ensembl.org/pub/current_gtf/rattus_norvegicus/Rattus_norvegicus.mRatBN7.2.112.chr.gtf.gz). Gene‐level quantification was performed using HTSeq v0.6.0,^[^ ^59^ ^]^ and gene expression levels were normalized and expressed as Fragments Per Kilobase Millon Mapped Reads (FPKM).

Differential gene expression analysis was conducted using DESeq2 v1.6.3.^[^ ^60^ ^]^ Genes with a *p* value < 0.05 and an absolute |log2FC| ≥ 1 were considered significantly differentially expressed. Functional enrichment analysis, including GO, KEGG, and Reactome pathway analysis, was performed. GO and KEGG enrichment were carried out using the clusterProfiler R package,^[^ ^61^ ^]^ while Reactome pathway analysis was conducted using the ReactomePA package.^[^ ^62^ ^]^ Enrichment was evaluated using a hypergeometric test, with a *p* value < 0.05 considered statistically significant. GO analysis included three categories: Biological Process (BP), Molecular Function (MF), and Cellular Component (CC). The Reactome pathway analysis was conducted by selecting the first 30 items according to the ascending order of *p* values.

### RT‐qPCR Analysis of Key Genes

The RNA concentration was assessed using a NanoDrop spectrophotometer (Thermo Scientific, Waltham, MA, United States). 1 µg of total RNA was used for complementary DNA synthesis using the HiScript IV RT SuperMix for qPCR (+gDNA wiper) Kit (Vazyme cat# R423‐01). Quantitative PCR was carried out on a Rotor GeneQ (Qiagen) cycler with ChamQ Universal SYBR qPCR Master Mix (Vazyme cat# Q711‐03) using primers for target genes and *β‐actin* as an internal control. Primers were designed for the following key genes based on transcriptomic enrichment analysis: *Ptgdr*, *Eln*, *Fam180a*, *Acta2*, *Cldn24*, and *Twist1*. All transcript levels were normalized to *β‐actin* mRNA using the 2^−ΔΔCT^ method and each PCR reaction was run in duplicate for each sample and repeated at least twice. The primer sequences are shown in Table S2 (Supporting Information).

### In Vitro Cytotoxicity and In Vivo Biocompatibility Assessments

Cytotoxicity assays were conducted using NIH‐3T3 and HUVEC cells. The viability of cells exposed to various treatments for 24 h was evaluated with the Calcein‐AM/PI cell viability/cytotoxicity assay kit. Additionally, the proliferation toxicity of PDA@ZIF‐8@AB NPs, PCL@QX‐314 MSs, and HA at different concentrations was assessed using the CCK‐8 assay. For in vivo biocompatibility evaluation, blood samples were collected from the orbital sinus of rats for hemolysis testing, and serum was obtained via centrifugation to analyze liver and kidney function. MN patches were inserted into the dorsal skin of rats, with careful monitoring for any signs of inflammation, redness, or swelling at the insertion sites. After a 7‐day period, skin and internal organs (heart, liver, spleen, lungs, and kidneys) were harvested and subjected to H&E staining to investigate potential inflammatory responses or tissue damage.

### Statistical Analysis

Statistical analysis was performed with data presented as mean ± standard error of the mean (SEM). All evaluations were conducted using GraphPad Prism 9 (GraphPad Software Inc., USA). Differences between two groups were analyzed using an unpaired two‐tailed Student's t‐test. For comparisons involving multiple groups, one‐way analysis of variance (ANOVA) followed by Tukey's post hoc test was employed. Equality of variances was assessed using Brown‐Forsythe test, and Welch's correction was applied if the assumption of equal variances was violated. A *p* value of less than 0.05 was considered statistically significant, with significance levels indicated as follows: ^*^ *p* < 0.05, ^**^ *p* < 0.01, ^***^ *p* < 0.001, and ^****^ *p* < 0.0001.

## Conflict of Interest

The authors declare no conflict of interest.

## Author Contributions

A.Zhang and X.Jiang. contributed equally to this work and share first authorship. W. Li and M. Peng designed and supervised the project. A. Zhang and X. Jiang designed the experimental strategies. A. Zhang, X. Jiang, B. Xiong, J. Chen, X. Liu, S. Wang and B. Li performed the experiments and analyzed the data. A. Zhang, W. Li, and M. Peng wrote the manuscript.

## Supporting information



Supporting Information

Supplemental Movie 1

Supplemental Movie 2

Supplemental Movie 3

Supplemental Movie 4

Supplemental Movie 5

Supplemental Movie 6

Supporting Tables

## Data Availability

The authors declare that all data supporting the findings of this study are available within the article and the Supplementary Information. All other data are available from the corresponding authors upon reasonable request.
