# Supplementary material for: Sustained‐Release Photothermal Microneedles for Postoperative Incisional Analgesia and Wound Healing via Hydrogen Therapy
Source: Adv Sci (Weinh). 2025 Jun 23;12(35):e03698. doi: 10.1002/advs.202503698 (PMC12463130; doi:10.1002/advs.202503698)
Supplement: Supplementary file 1 — Supporting Information [file ADVS-12-e03698-s004.docx]

**Supplementary Figure Legends**

**
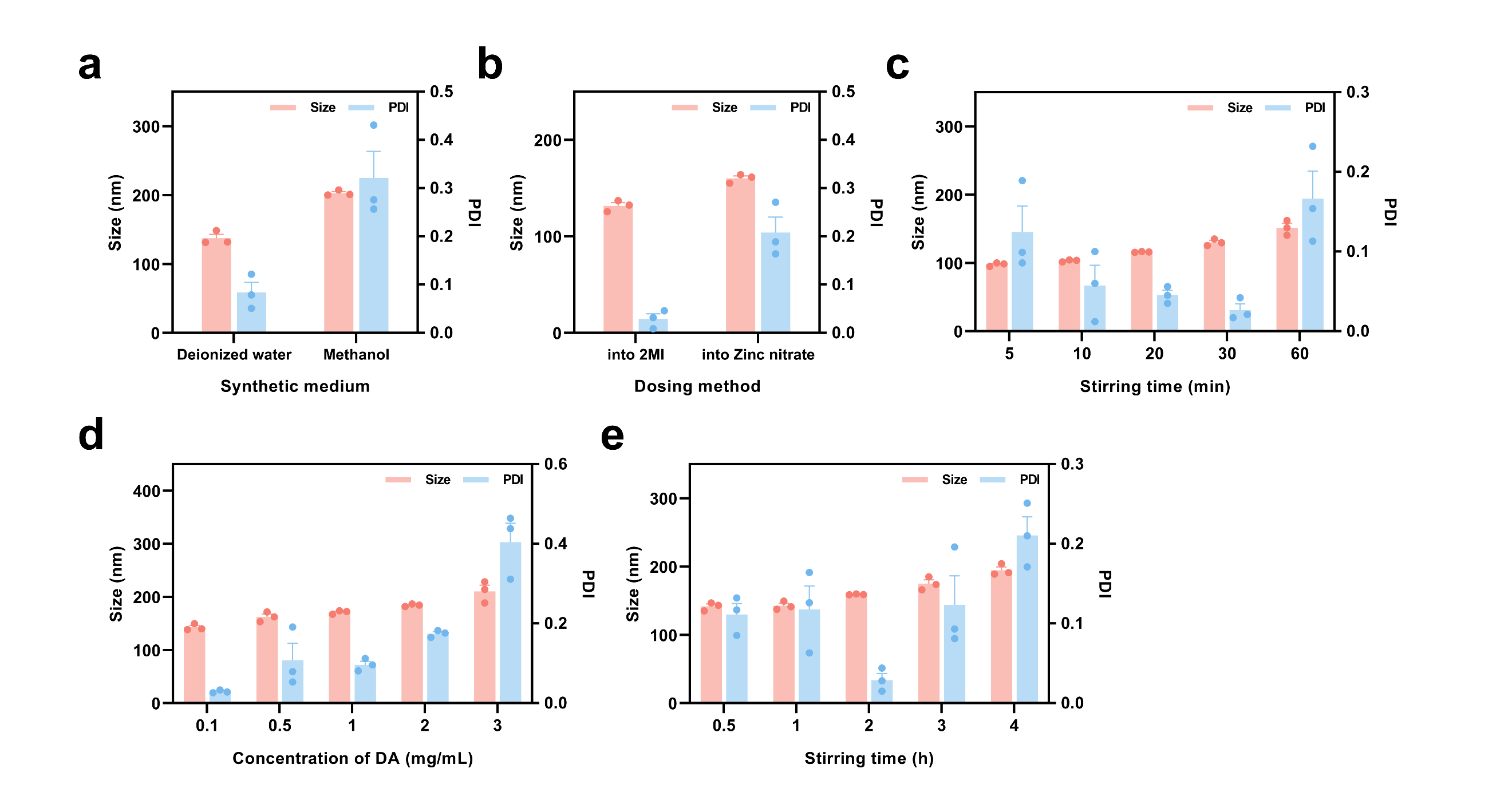
**

**Supplementary Figure 1. Optimization of synthesis parameters for PDA@ZIF-8@AB NPs.**

**a** Effect of synthetic medium on particle size and PDI. **b** Comparison of AB addition methods (into 2-MI vs zinc nitrate solution) on particle characteristics. **c** Influence of stirring time on particle size and PDI. **d** Effect of DA concentration on particle size and PDI. **e** Influence of prolonged stirring on particle characteristics. Data were presented as mean ± SEM from n = 3 independent experiments. PDI: polydispersity index. 2-MI: 2-Methylimidazole.

**
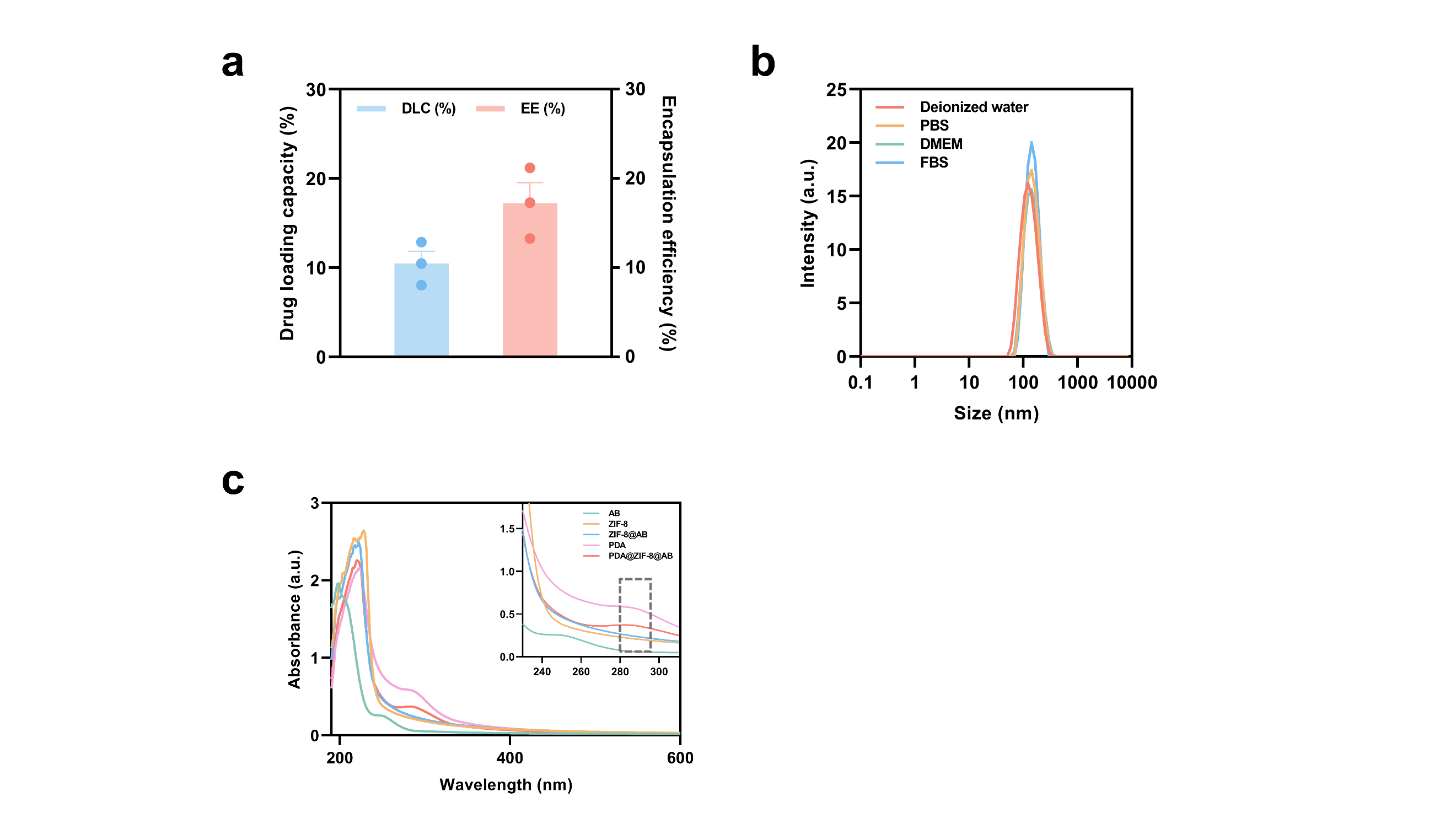
**

**Supplementary Figure 2. Drug loading capacity, encapsulation efficiency, particle stability, and UV–vis absorbance spectra of PDA@ZIF-8@AB NPs.**

**a** Drug loading capacity and encapsulation efficiency of PDA@ZIF-8@AB NPs. **b** Particle size stability of ZIF-8@AB NPs after 24 hours in different media (deionized water, PBS, DMEM, and FBS). **c** UV-vis absorbance spectra of different materials. Data were presented as mean ± SEM from n = 3 independent experiments.

**
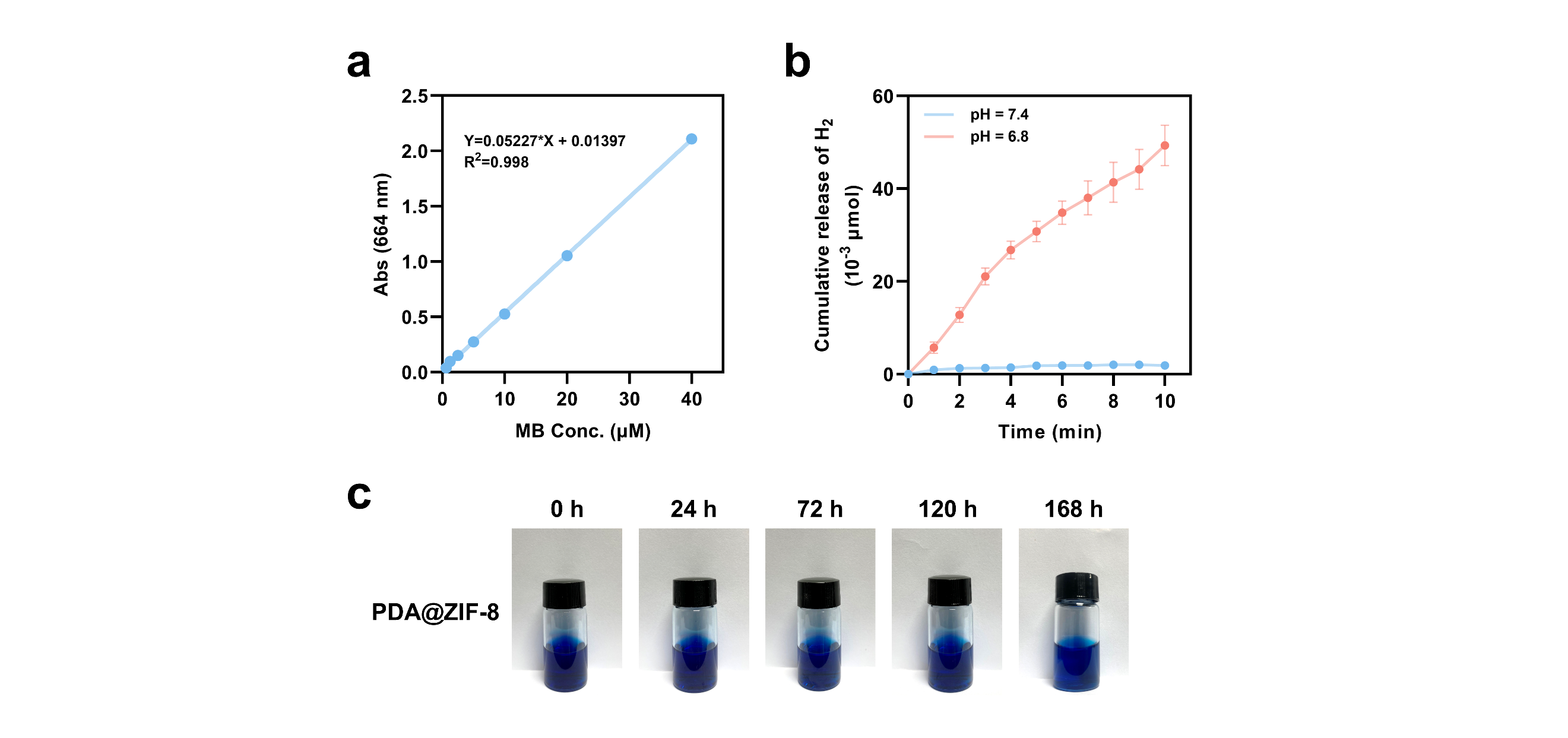
**

**Supplementary Figure 3. H_2_ release characterization of AB and PDA@ZIF-8 NPs.**

**a** Standard curve of MB concentration against absorbance at 664 nm. **b** Cumulative H_2_ release profiles of free AB at pH 7.4 and pH 6.8 over 10 minutes. **c** Stability test of PDA@ZIF-8 NPs in MB-Pt probe solution over 168 hours, showing visual changes at different time points. Data were presented as mean ± SEM from n = 3 independent experiments.

**
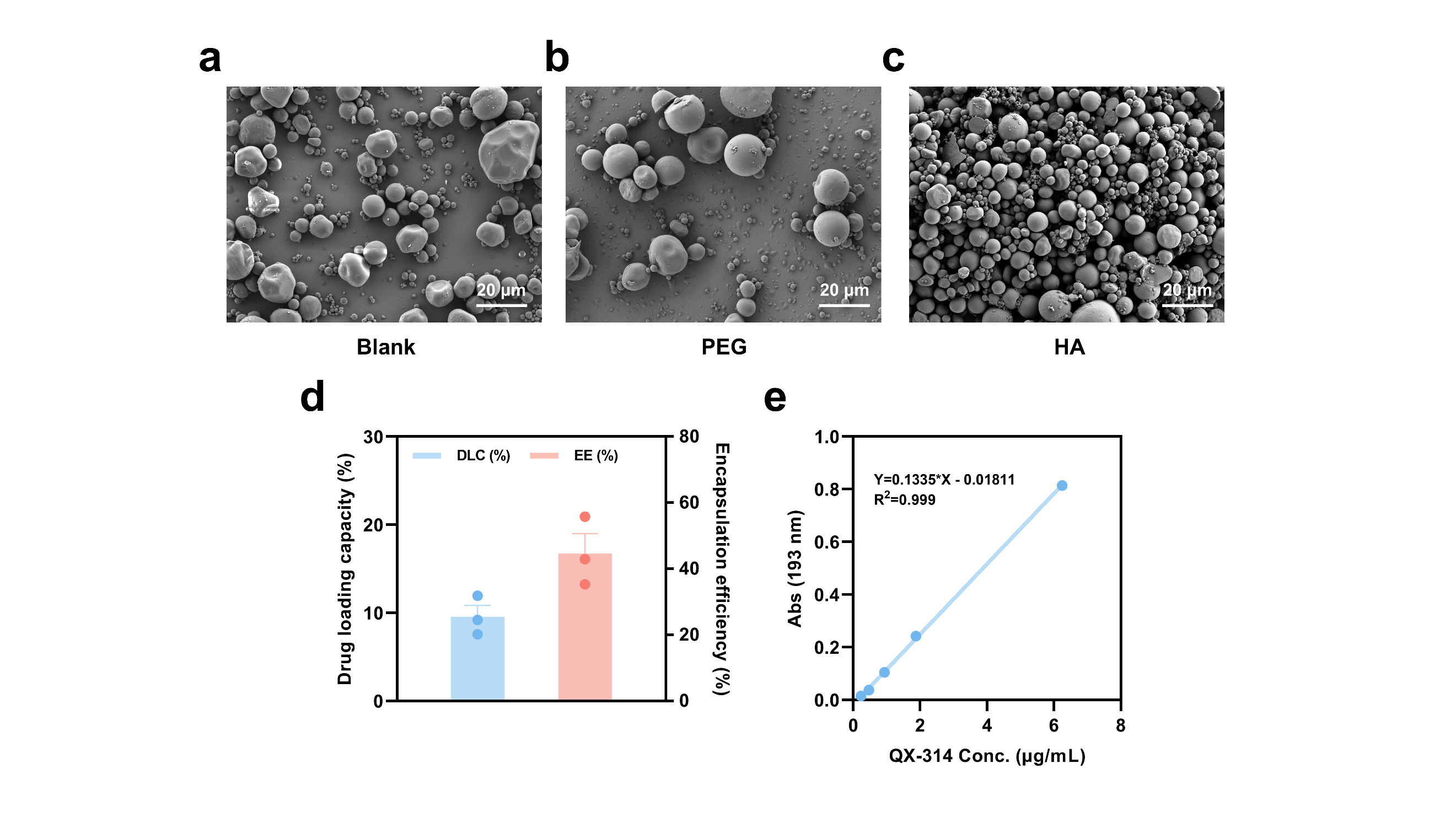
**

**Supplementary Figure 4. Characterization of PCL@QX-314 MSs with different stabilizers and QX-314 loading.**

**a–c** SEM images showing the surface morphology of PCL@QX-314 MSs prepared with different stabilizers: Blank **(a)**, PEG (**b**), and HA (**c**). **d** Drug loading capacity and encapsulation efficiency of PCL@QX-314 MSs. **e** Standard curve of QX-314 concentration against absorbance at 193 nm. Data were presented as mean ± SEM from n = 3 independent experiments.

**
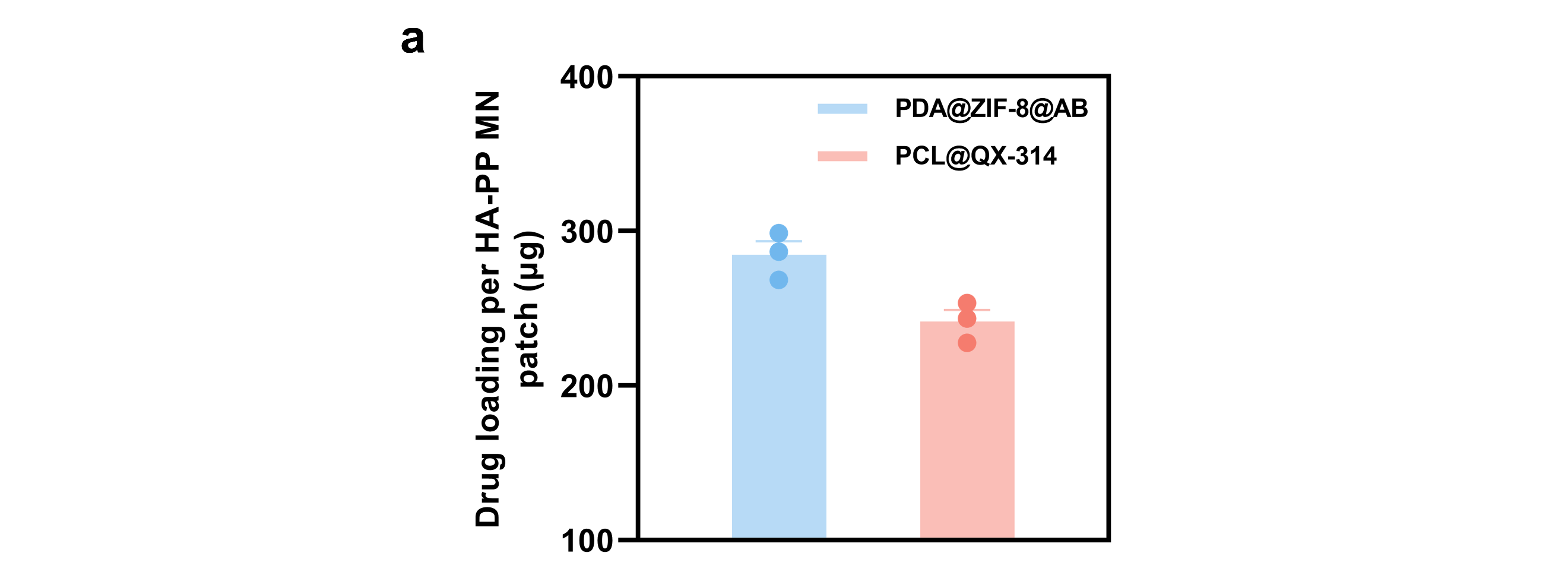
**

**Supplementary Figure 5. Drug loading per HA-PP MN patch.**

**a** Drug loading capacity of PDA@ZIF-8@AB NPs and PCL@QX-314 MSs in each HA-PP MN patch. Data were presented as mean ± SEM from n = 3 independent experiments.

**
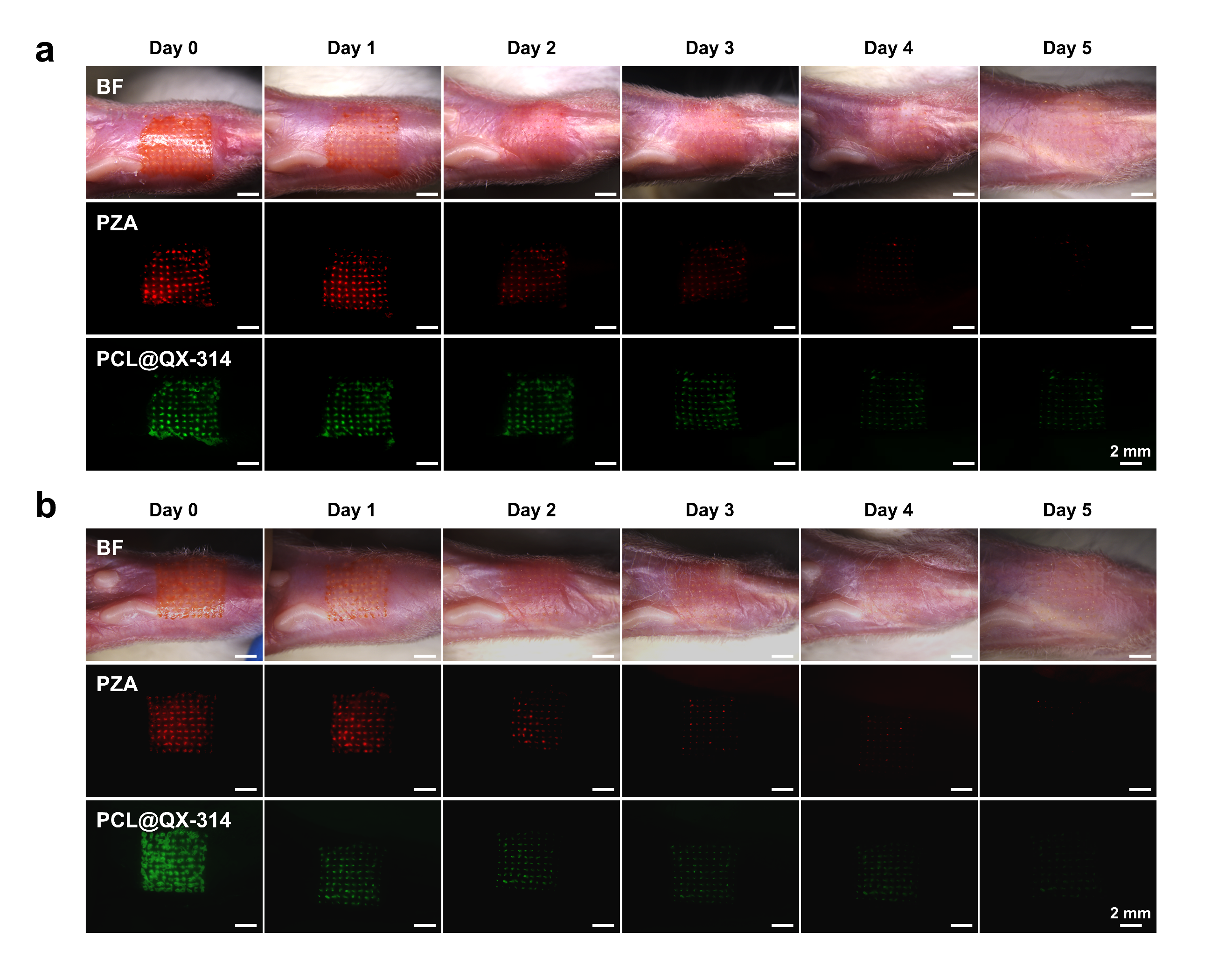
**

**Supplementary Figure 6. Retention and drug release of PDA@ZIF-8@AB NPs and PCL@QX-314 MSs after in vivo application of HA-PP MN patch.**

**a Images showing the local retention and release of PDA@ZIF-8@AB NPs and PCL@QX-314 MSs in the acidic environment of the rat paw skin without NIR irradiation. b Images showing the local retention and release of PDA@ZIF-8@AB NPs and PCL@QX-314 MSs in the acidic environment of the rat paw skin with daily NIR irradiation (5 min).**

**
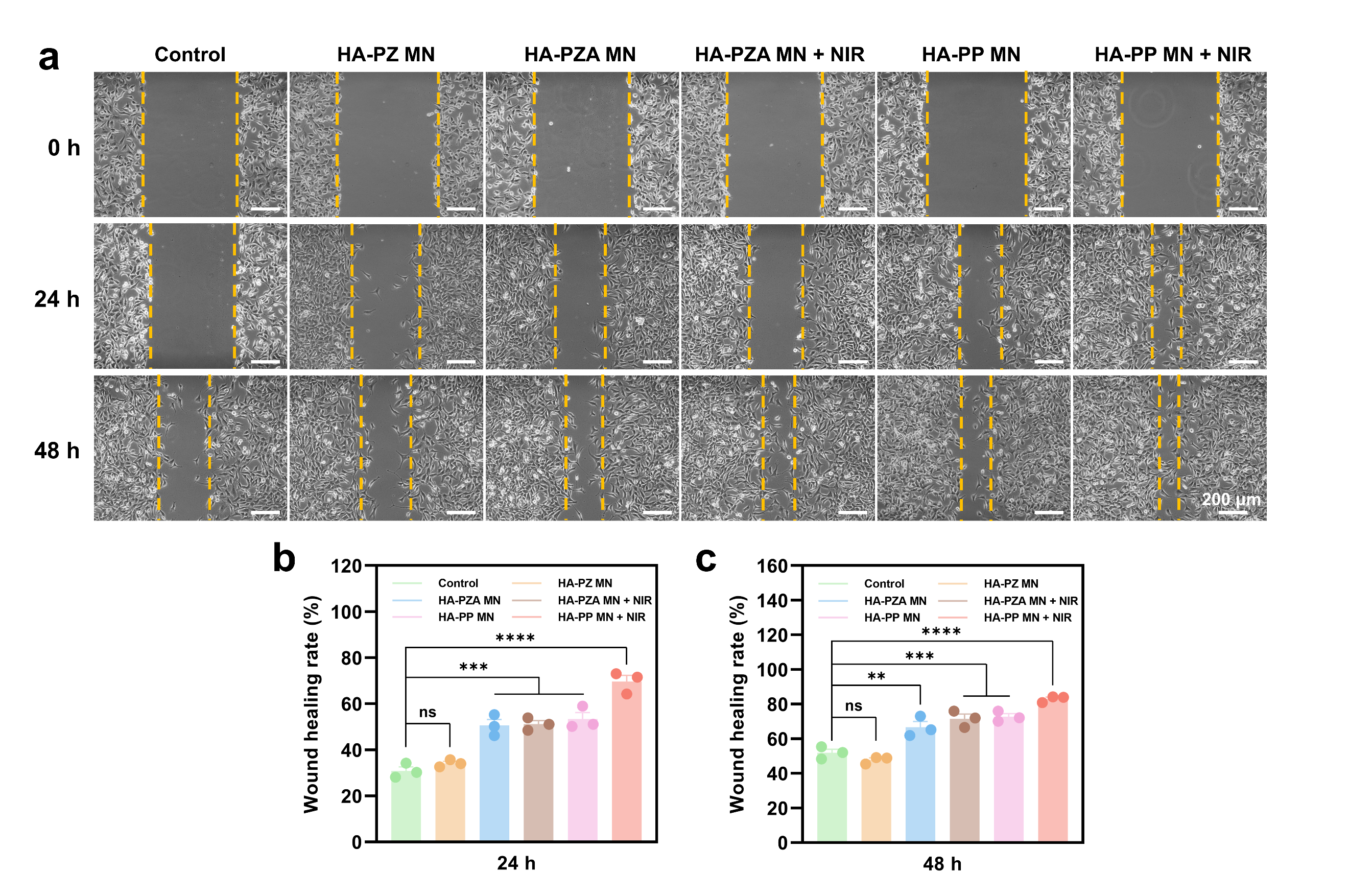
**

**Supplementary Figure 7. Evaluation of wound healing of various MN patches on NIH-3T3 cells.**

**a** Bright field images showing the scratch assay of NIH-3T3 cells treated with different MN patches at 0, 24, and 48 hours. **b** Quantification of wound healing rate at 24 hours for each treatment group. **c** Quantification of wound healing rate at 48 hours for each treatment group. Data were presented as mean ± SEM from n = 3 independent experiments. *P* value: ns means no significance, ^**^*p* < 0.01, ^***^*p* < 0.001, and ^****^*p* < 0.0001.

**
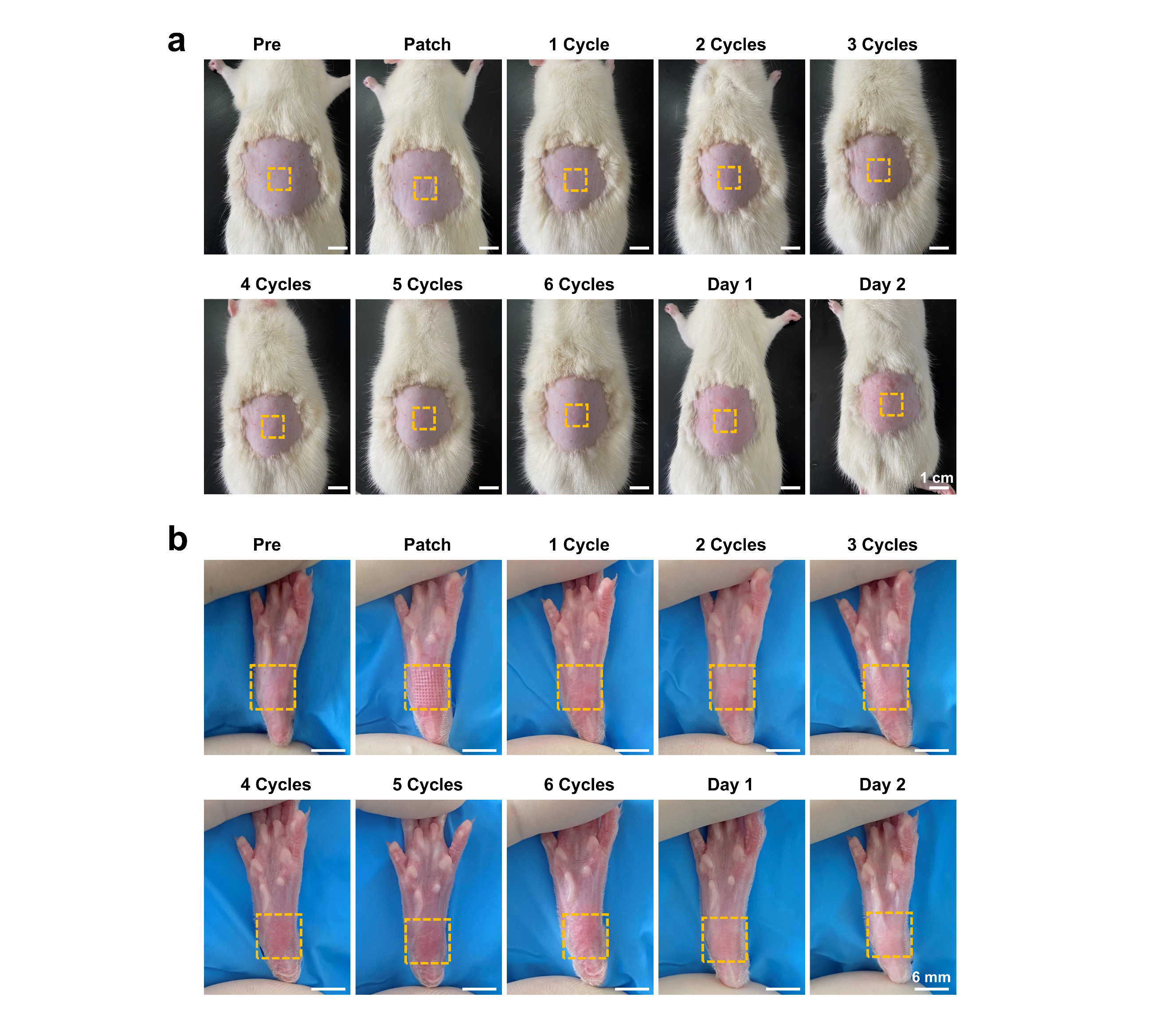
**

**Supplementary Figure 8. Effects of multiple NIR irradiations on skin after HA-PZA MN patch application in rat models.**

**a** Representative images of the dorsal skin of rats showing the application area of the HA-PZA MN patch across various NIR irradiation cycles and post-removal time points (Day 1 and Day 2). **b** Representative images of the hind paw skin of rats showing the application area of the HA-PZA MN patch across various NIR irradiation cycles and post-removal time points (Day 1 and Day 2).

**
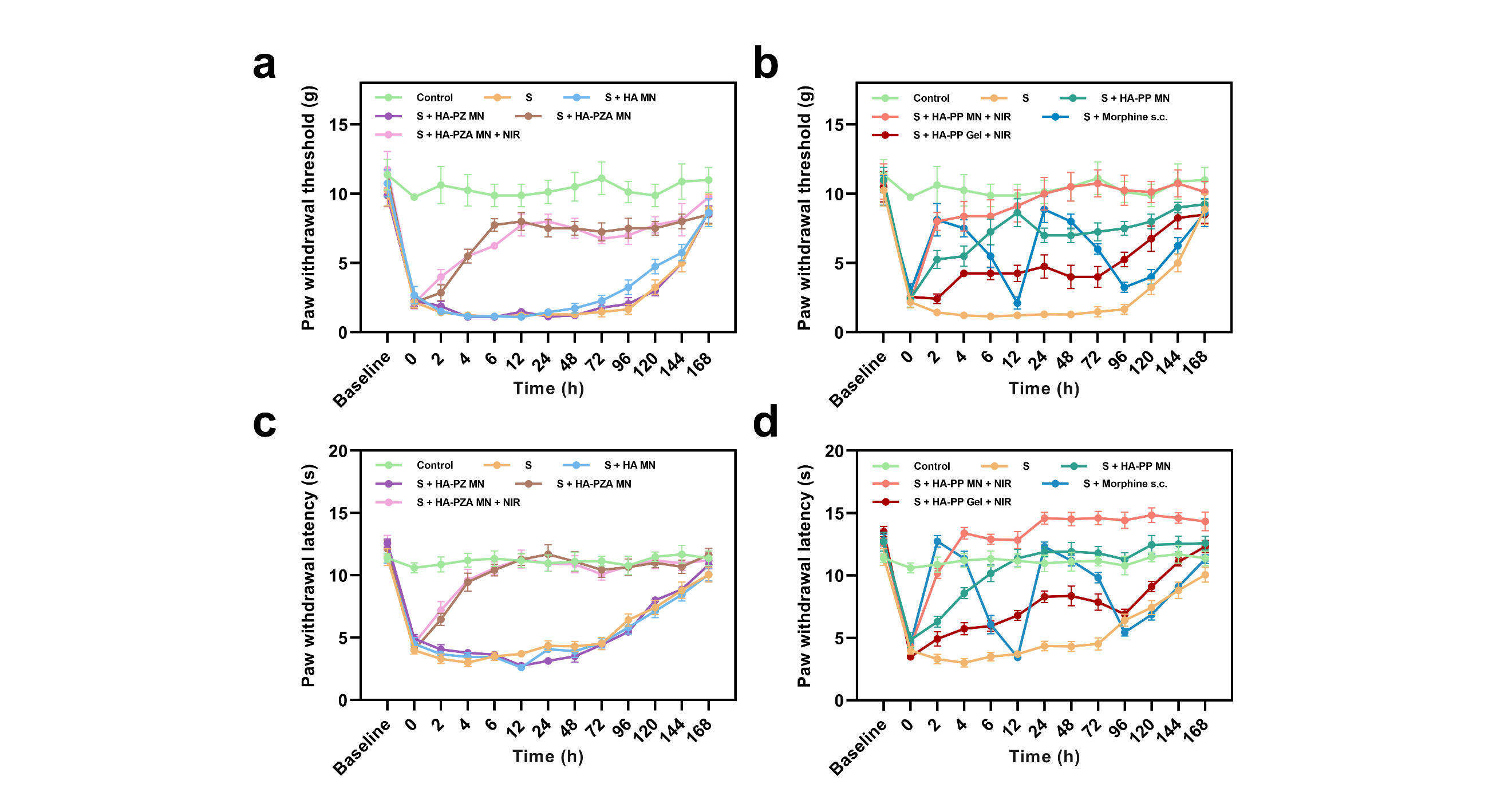
**

**Supplementary Figure 9. Behavioral tests for pain assessment following plantar incision surgery in rats and the effects of various MN treatments.**

**a-b** Mechanical paw withdrawal threshold measurements before and after MN treatments. **c-d** Thermal paw withdrawal latency measurements before and after MN treatments. All data were expressed as mean ± SEM (n = 8).

**
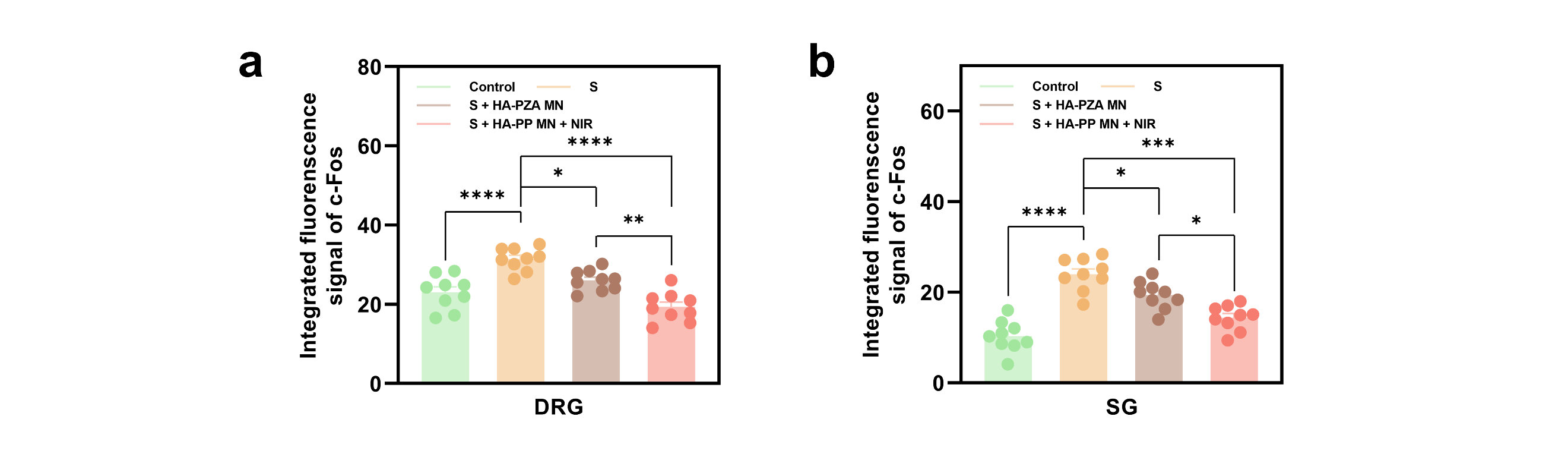
**

**Supplementary Figure 10. Quantitative analysis of c-Fos expression in DRG and SG area of spinal cord dorsal horn following MN treatments.**

**a** Integrated fluorescence intensity of c-Fos in DRG across different treatment groups. **b** Integrated fluorescence intensity of c-Fos in the SG area of spinal cord dorsal horn. All data were expressed as mean ± SEM (n = 9 slices from three rats). *P* value: ^*^*p* < 0.05, ^**^*p* < 0.01, ^***^*p* < 0.001, and ^****^*p* < 0.0001.

**
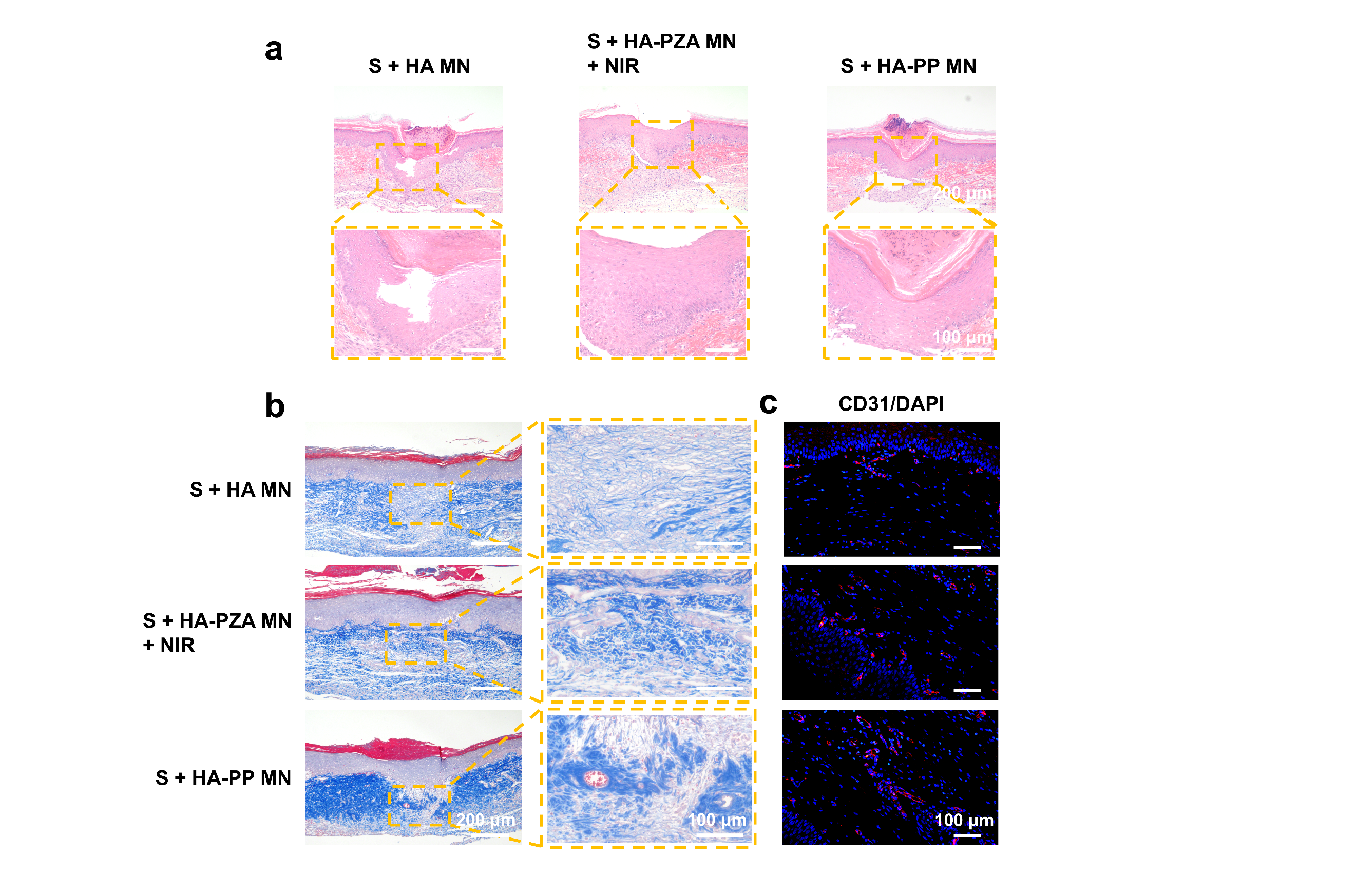
**

**Supplementary Figure 11. Evaluation of wound healing, collagen deposition, and angiogenesis following MN treatments.**

**a** H&E-stained tissue sections of the wound area. **b** Masson’s trichrome staining for collagen fibers in wound tissues. **c** Immunofluorescence staining for CD31 in wound sections to assess angiogenesis.

**
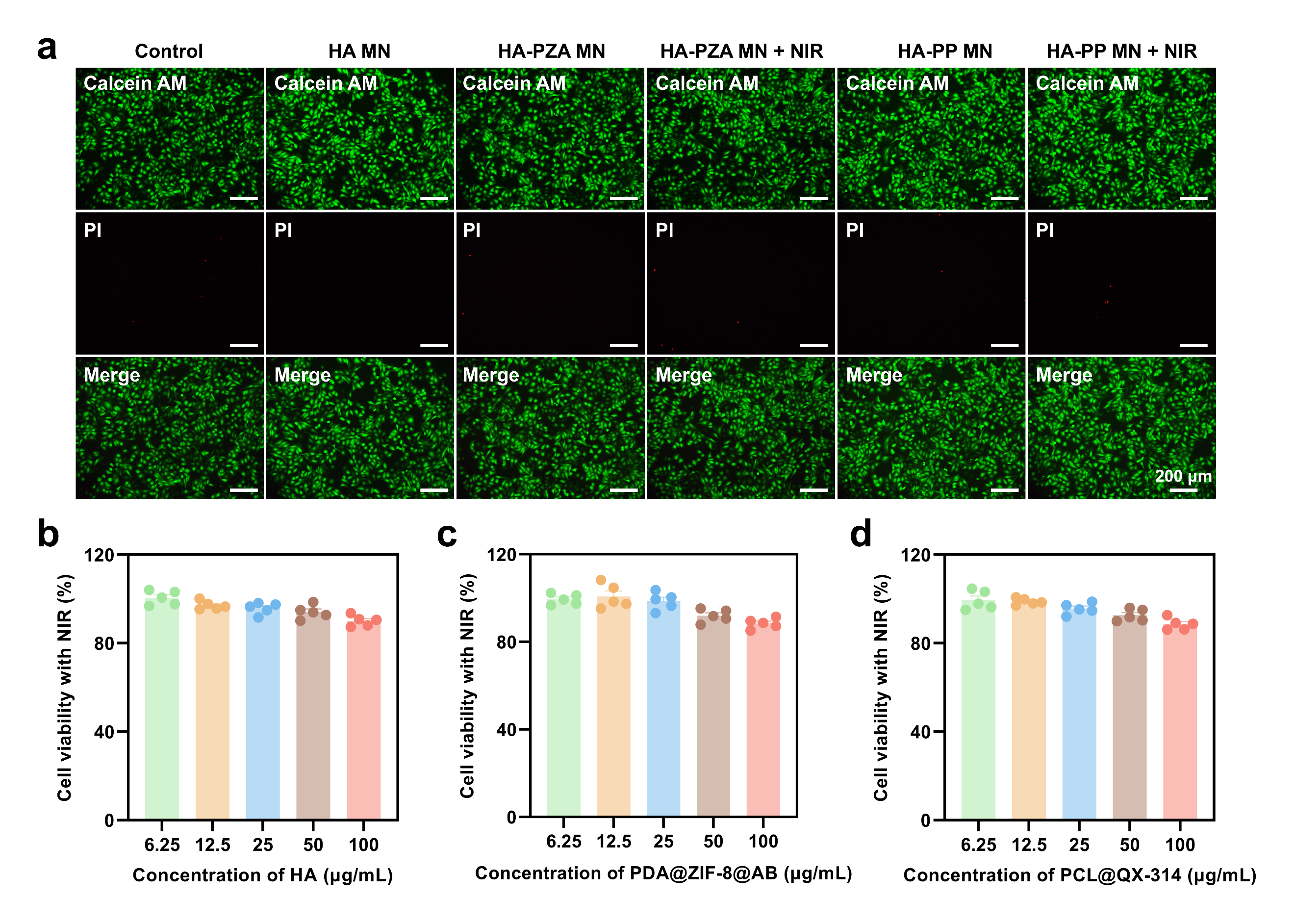
**

**Supplementary Figure 12. Cytotoxicity of various MN patches on HUVEC cells with NIR irradiation.**

**a** Live/Dead staining images of HUVEC cells treated with different MN patches. Green fluorescence (Calcein-AM) indicated live cells, while red fluorescence (PI) represented dead cells. **b-d** Cell viability of HUVECs exposed to increasing concentrations of HA **(b)**, PDA@ZIF-8@AB NPs **(c)**, and PCL@QX-314 MSs **(d)** in the presence of NIR irradiation. Data were presented as mean ± SEM from n = 5 independent experiments.

**
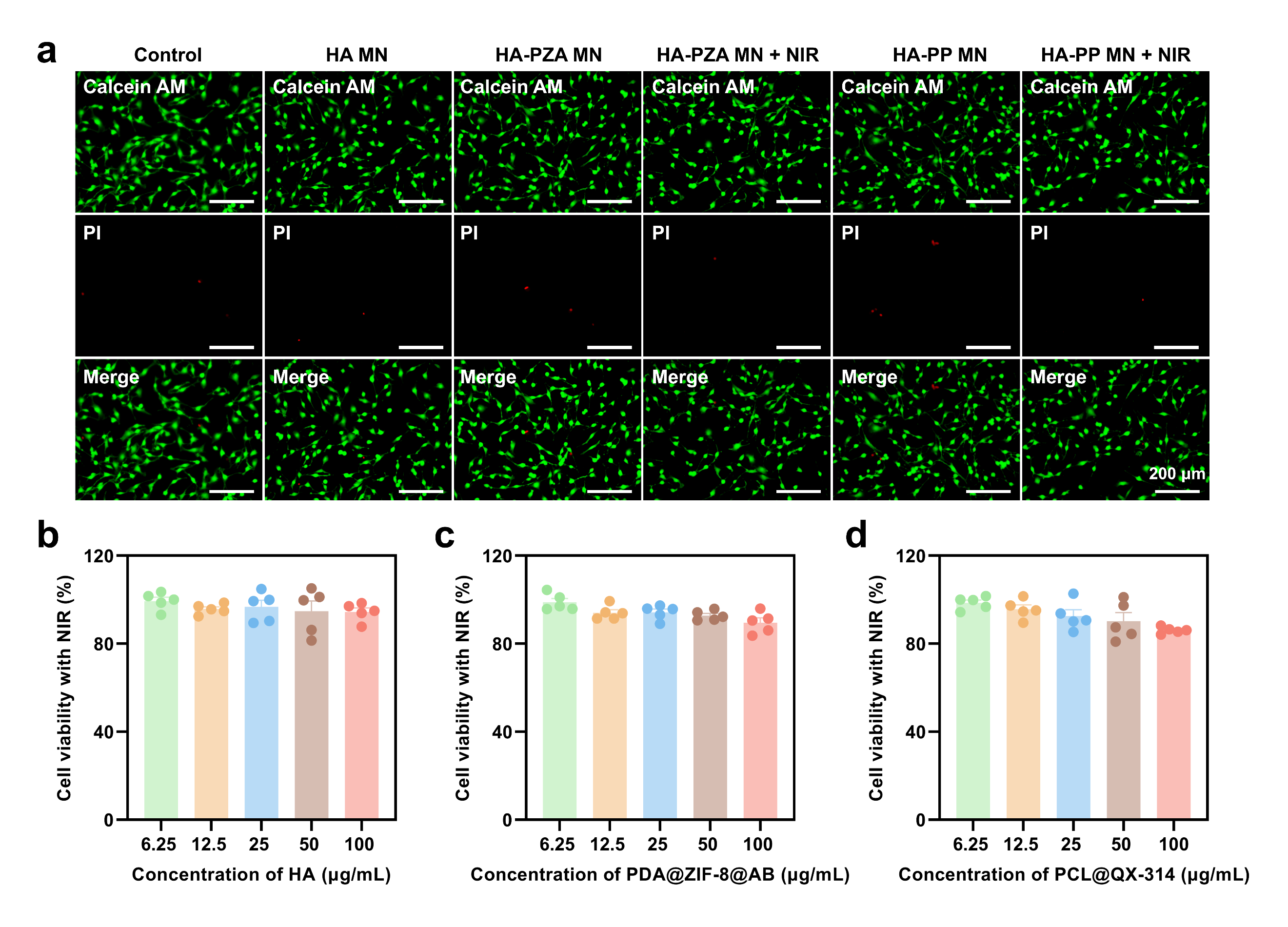
**

**Supplementary Figure 13. Cytotoxicity of various MN patches on** **NIH-3T3 cells with NIR irradiation.**

**a** Live/Dead staining images of NIH-3T3 cells treated with different MN patches. Green fluorescence (Calcein-AM) indicated live cells, while red fluorescence (PI) represented dead cells. **b-d** Viability of NIH-3T3 cells exposed to increasing concentrations of HA **(b)**, PDA@ZIF-8@AB NPs **(c)**, and PCL@QX-314 MSs **(d)** in the presence of NIR irradiation. Data were presented as mean ± SEM from n = 5 independent experiments.

**
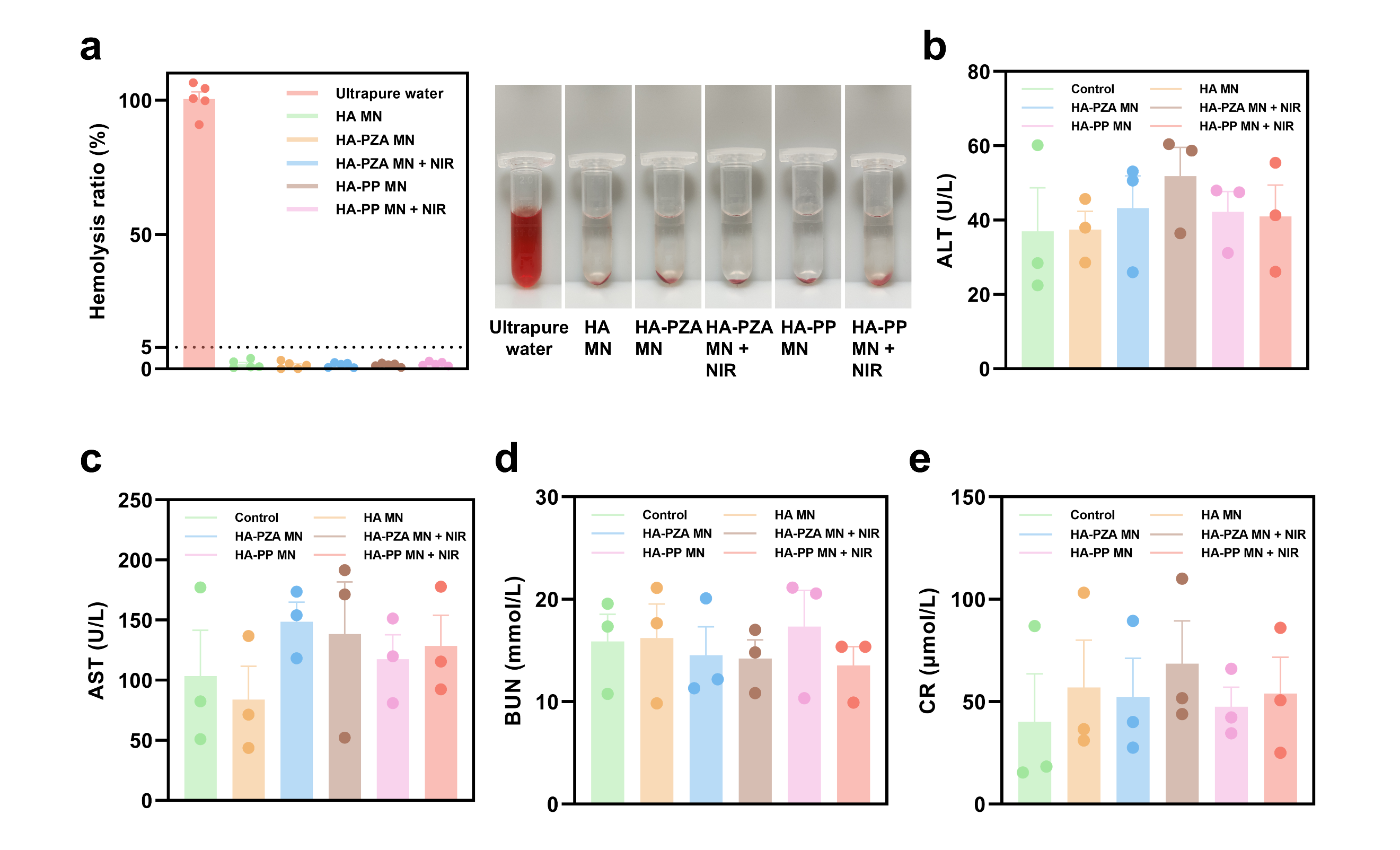
**

**Supplementary Figure 14. In vivo biocompatibility evaluation of MN patches by hemolysis assay and serum biochemical markers.**

**a** Hemolysis assay results for different MN patches. Right panel: photographs of hemolysis assay tubes with different MN treatments. **b-e** Serum biochemical markers in different treated rats: ALT (**b**), AST (**c**), BUN (**d**), and CR (**e**). Data were presented as mean ± SEM from n = 5 or n = 3 independent experiments.

**
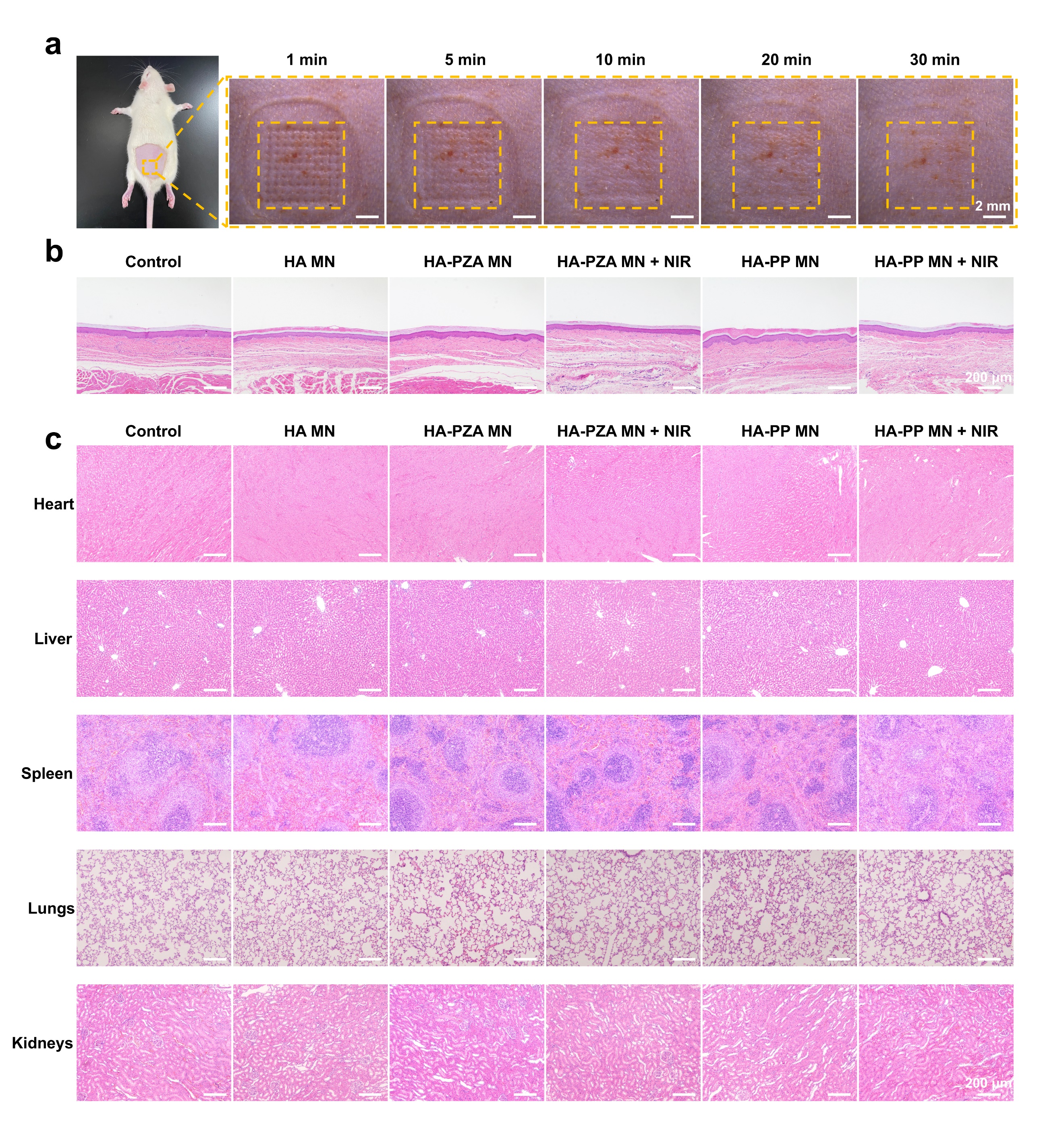
**

**Supplementary Figure 15. Micropore healing and histopathological assessment of MN patches.**

**a** Representative images of the application area on rat skin after exposure to HA-PP MN with NIR irradiation at different time points. **b** H&E-stained sections of skin tissue with different treatments. **c** Histological examination of major organs (heart, liver, spleen, lungs, and kidneys) from different treated rats.
