# Supplementary material for: Sustained‐Release Photothermal Microneedles for Postoperative Incisional Analgesia and Wound Healing via Hydrogen Therapy
Source: Adv Sci (Weinh). 2025 Jun 23;12(35):e03698. doi: 10.1002/advs.202503698 (PMC12463130; doi:10.1002/advs.202503698)
Supplement: Supplementary file 8 — Supporting Tables [file ADVS-12-e03698-s001.docx]

**Supplementary Table 1. RNA-seq mapping rates across different groups.**

| Group/Sample | Control | | | Surgery | | | Treatment | | |
| --- | --- | --- | --- | --- | --- | --- | --- | --- | --- |
|  | C1 | C2 | C3 | S1 | S2 | S3 | T1 | T2 | T3 |
| Mapping  rate (%) | 98.06 | 97.85 | 97.73 | 97.92 | 98.06 | 98.04 | 97.81 | 97.89 | 97.80 |

**Supplementary Table 2. Primer sequences used in RT-qPCR.**

| **Gene name** | **Forward primer** | **Reverse primer** |
| --- | --- | --- |
| *β-actin* | CCCATCTATGAGGGTTACGC | TTTAATGTCACGCACGATTTC |
| *Ptgdr* | TTCCCTGCCTTTAATTTATCGTGC | AGGGGTCCACGATGGAAATC |
| *Eln* | GCAGCGAAGTATGGTGCTGG | TGGGACCCTAACTCCTGGTC |
| *Fam180a* | ATTCGAAGGCTGAGTGCCAA | ATCAAGTCATGCCTCAGGGC |
| *Acta2* | CAGCTATGTGGGGGACGAAG | TCCGTTAGCAAGGTCGGATG |
| *Cldn24* | AAAACGGCCATGCAATCGG | GGATGACGCAGGATTTCCAGA |
| *Twist1* | TCGGACAAGCTGAGCAAGATT | GCAGCTTGCCATCTTGGAGT |
